# Spatiotemporal patterns and association with climate for malaria elimination in Lao PDR: a hierarchical modelling analysis with two-step Bayesian model selection

**DOI:** 10.1186/s12936-024-05064-0

**Published:** 2024-08-04

**Authors:** Chawarat Rotejanaprasert, Vilayvone Malaphone, Mayfong Mayxay, Keobouphaphone Chindavongsa, Virasack Banouvong, Boualam Khamlome, Phoutnalong Vilay, Viengxay Vanisavaeth, Richard J. Maude

**Affiliations:** 1 Department of Tropical Hygiene, Faculty of Tropical Medicine, Mahidol University, Bangkok, Thailand; 2 Mahidol-Oxford Tropical Medicine Research Unit, Faculty of Tropical Medicine, Mahidol University, Bangkok, Thailand; 3 Institute for Research and Education Development, University of Health Sciences, Vientiane, Lao PDR; 4 Lao-Oxford-Mahosot Hospital-Wellcome Trust Research Unit, Microbiology Laboratory, Mahosot Hospital, Vientiane, Lao PDR; 5 Centre for Tropical Medicine and Global Health, Nuffield Department of Clinical Medicine, University of Oxford, Oxford, UK; 6 Saw Swee Hock School of Public Health, National University of Singapore, Singapore, Singapore; 7 Center of Malariology, Parasitology, and Entomology, Vientiane, Lao PDR; 8 The Open University, Milton Keynes, UK

**Keywords:** Malaria, Spatiotemporal, Climate, Lao PDR, Hierarchical modeling

## Abstract

**Background:**

The government of Lao PDR has increased efforts to control malaria transmission in order to reach its national elimination goal by 2030. Weather can influence malaria transmission dynamics and should be considered when assessing the impact of elimination interventions but this relationship has not been well characterized in Lao PDR. This study examined the space–time association between climate variables and *Plasmodium falciparum* and *Plasmodium vivax* malaria incidence from 2010 to 2022.

**Methods:**

Spatiotemporal Bayesian modelling was used to investigate the monthly relationship, and model selection criteria were used to evaluate the performance of the models and weather variable specifications. As the malaria control and elimination situation was spatially and temporally dynamic during the study period, the association was examined annually at the provincial level.

**Results:**

Malaria incidence decreased from 2010 to 2022 and was concentrated in the southern regions for both *P. falciparum* and *P. vivax*. Rainfall and maximum humidity were identified as most strongly associated with malaria during the study period. Rainfall was associated with *P. falciparum* incidence in the north and central regions during 2010–2011, and with *P. vivax* incidence in the north and central regions during 2012–2015. Maximum humidity was persistently associated with *P. falciparum* and *P. vivax* incidence in the south.

**Conclusions:**

Malaria remains prevalent in Lao PDR, particularly in the south, and the relationship with weather varies between regions but was strongest for rainfall and maximum humidity for both species. During peak periods with suitable weather conditions, vector control activities and raising public health awareness on the proper usage of intervention measures, such as indoor residual spraying and personal protection, should be prioritized.

**Supplementary Information:**

The online version contains supplementary material available at 10.1186/s12936-024-05064-0.

## Background

The Greater Mekong Sub-region (GMS) has experienced a significant decline in malaria burden over the past decade. However, facing the growing threat of anti-malarial drug resistance, governments in the region have committed to eliminating malaria by 2030. In Lao PDR, the government aims to eliminate malaria in the north by 2025 and in the entire country by 2030, with an interim evaluation of strategies planned for 2023. Malaria transmission is sensitive to weather and environment, varying in degree depending on the local entomology and epidemiology (see examples [1–4]). The Lao government is interested in exploring the local relationship between weather and malaria at different spatial and temporal scales to better understand the impact of their elimination activities. This exploration may inform the optimization of resource allocation and the development of more effective strategies to accelerate progress towards elimination.

Several methods have been proposed in the spatiotemporal framework to select potential models or linear predictors of interest. Given the complexity of malaria transmission, hierarchical model selection is perhaps more suitable than simple variable selection [5]. Selecting the appropriate model or linear predictors is a crucial aspect of epidemiological investigation, which can be challenging when data involve spatial and temporal dimensions. To address this challenge, various approaches such as model averaging and transformation selection have been suggested and applied to accomplish these objectives (see examples in [5–7]). For the purpose of investigating the spatiotemporal climatic association, examining different hierarchical spatiotemporal model specifications of retrospective malaria incidence data can be beneficial to the national malaria programme to properly plan future disease control and elimination interventions.

**Fig. 1:**
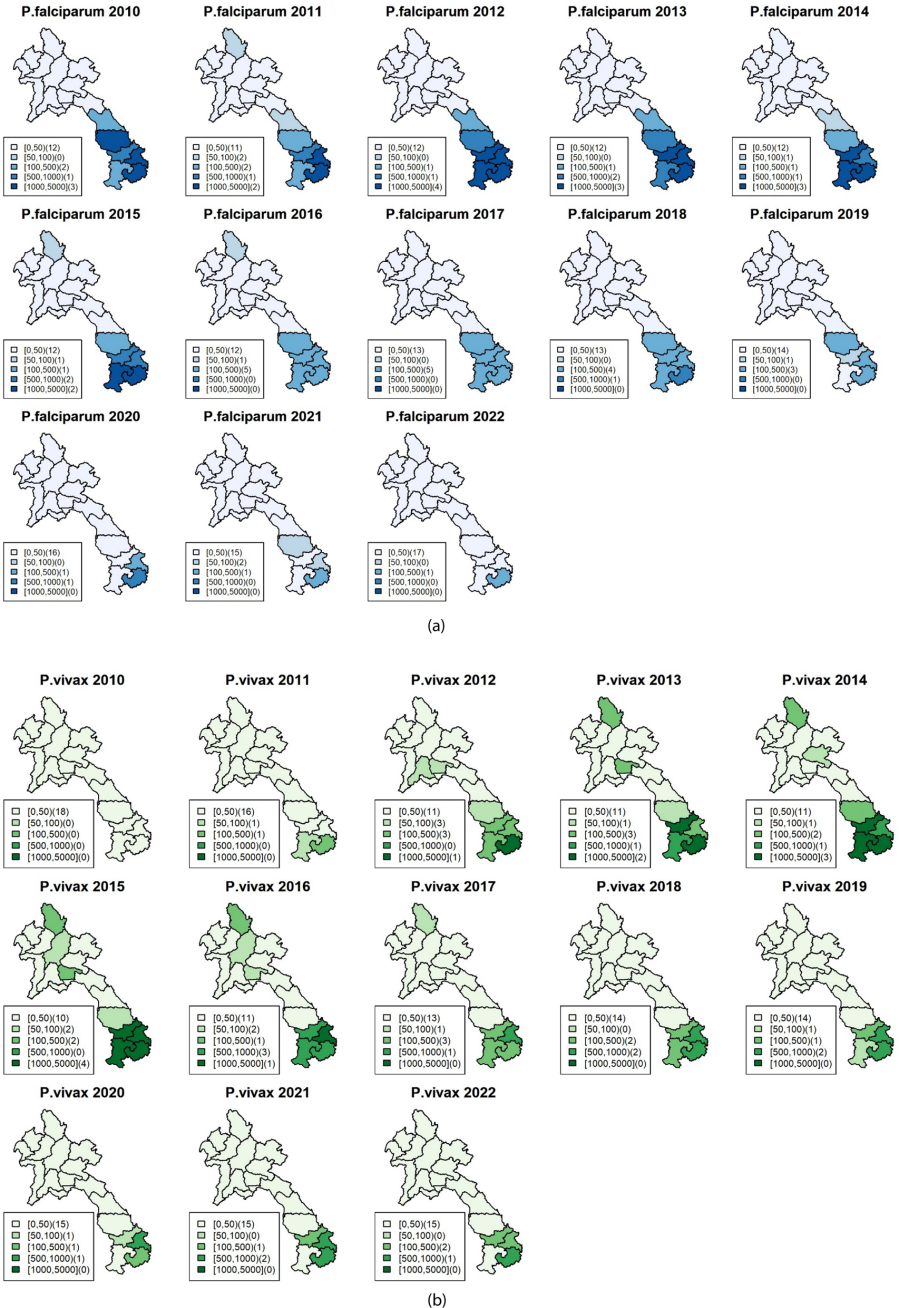
**a** Maps of provincial malaria incidence per 100,000 population of *Plasmodium falciparum* in Lao PDR during 2010–2022. **b** Maps of provincial malaria incidence per 100,000 population of *Plasmodium vivax* in Lao PDR during 2010–2022

In Lao PDR, national malaria elimination strategies have been planned and implemented since 2011, resulting in a substantial decrease in national annual parasite incidence. However, in recent years, malaria has become more geographically confined to focal areas in the country. Specifically, incidence has been low and sporadic in the north, whereas a higher burden is observed in the south, accounting for the majority of malaria cases in the country (refer to Fig. 1). The national strategic plan for malaria control and elimination during 2016–2020 focused on reducing disease transmission in the five provinces in the south, while the 13 northern provinces were targeted for elimination [8]. To implement effective malaria control and elimination activities, a good understanding of transmission dynamics in both space and time is required [9]. Therefore, spatiotemporal analysis is necessary to re-assess the impact of control and elimination measures subnationally in interim evaluations of progress towards malaria elimination goals [10].

The aim of this study was to investigate and describe space–time malaria patterns by species at the provincial level in Lao PDR and to quantify the association with climatic variables. Spatiotemporal Bayesian analyses were conducted using two-step model selection techniques. These methods allow us to analyse the effect of climatic factors simultaneously on the distribution of malaria incidence, while also considering space and time dependencies. Previous malaria studies have demonstrated the benefits of Bayesian methodologies in modeling malaria transmission and quantifying its spatiotemporal association with environmental factors at different scales (see examples [2, 3, 11]). This study focused on modeling malaria incidence at the provincial scale in Lao PDR, utilizing monthly climatic data from climate stations from 2010 to 2022. The findings can be useful in evaluating whether weather information can feasibly be used routinely to guide malaria surveillance and control activities.

## Methods

### Study design and data sources

This study utilized spatiotemporal retrospective analysis of surveillance data collected between 2010 and 2022 in Lao PDR. Malaria is a notifiable infectious disease in Lao PDR, and passive malaria surveillance is carried out by various levels of health facilities. Malaria cases recorded at health facilities have been reported into the District Health Information System 2 (DHIS2), which allows tracking of infected cases. Monthly provincial malaria case counts of clinically diagnosed malaria were obtained from DHIS2, Center for Malaria Parasitology and Entomology of Lao PDR (CMPE), from January 2010 to December 2022. Weather data for each province during the study period were sourced from the Department of Meteorology and Hydrology and the Department of Natural Resources and Environment, Lao PDR. Meteorological data were obtained from a weather station located in the provincial capital cities. The minimum and maximum temperatures (in Celsius), minimum and maximum relative humidity (in percent), and rainfall (in millimetres) at the provincial level were calculated by averaging daily data and aggregating them into monthly data.

### Spatiotemporal hierarchical modelling formulation

Modelling the potential associations between spatial and temporal variability in malaria incidence and climatic variables can be approached in various ways. In Frequentist modelling, negative binomial regression is often used for overdispersed count data, though other models are also available. In the context of Bayesian regression models for count data, spatial dependence can be addressed by specifying a hierarchical model, which includes a set of random effects in the linear predictor. The study incorporated and compared different space–time random effect structures. The literature in spatial epidemiology and surveillance has extensively examined the performance and handling of overdispersion using various count models, including Poisson, negative binomial, and generalized Poisson models. These studies suggest that while models like the negative binomial can handle overdispersion, Poisson models with space–time random effects can perform similarly well and better in some cases (see examples [12–14]).

Space–time hierarchical modelling is an approach that incorporates similarities between incidence data observed at different locations, allowing for borrowing strength across spatial units. Additionally, the temporal aspect of the modelling approach permits inference concerning temporal trends of changes in malaria cases. This modelling framework can effectively capture the spatial and temporal variation in the malaria data, enabling the quantification of the association with environmental variables. By addressing spatial and temporal dependencies, this approach can provide a comprehensive understanding of the climate-malaria relationship, which is important for informing public health interventions and policy decisions.

To model the malaria cases, let $y_{it}$ be the number of new malaria cases for each species in province* i* and at month *t* where I and T were the total number of spatial (18 provinces) and temporal (156 months of collected data) units in the study. Since the health outcome of interest is in the form of case count data, a common likelihood for hierarchical modeling is the Poisson model (see examples [15–18]) as $y_{it} \sim Poisson(\mu_{it} )$ where $\mu_{it}$ is the mean malaria incidence in each province and month. To model the mean incidence, the natural logarithm was used as the canonical link function as $\log (\mu_{it} ) = \log (offset) + \log (\eta_{it} )$, where $\eta_{it}$ is the linear predictor. The offset here was applied to handle different susceptibilities of malaria transmission across space–time units. There are a number of ways to calculate the offset; the number of populations at risk was used in our study, i.e., intensity is the ratio of mean number of cases and population. Then, the predictor $\eta_{it}$ can be decomposed as a linear combination of fixed climatic and space–time random effects as $\eta_{it} = {\varvec{X}}_{it}^{T} {\varvec{\beta}} + \xi_{i} + \lambda_{t} + \theta_{it}$ where ${\varvec{X}}_{it}^{{}}$ is a design matrix of province-level predictors;${\varvec{\beta}}$ is a vector of regression coefficients for each environmental variable; $\xi_{i}$ and $\lambda_{t}$ are the conditional spatial and temporal random effects of area *i* and time period *t*, respectively; and $\theta_{it}$ denotes a corresponding space–time interaction. To estimate the parameters, a fully Bayesian framework was adopted in which a prior distribution needs to be specified for all parameters in the model.

The random effects were spatially structured by borrowing information across neighbouring regions and time periods to incorporate spatiotemporal smoothing. The convolution model was modelled to the spatial random effect as $\xi_{\tau ,i} = u_{i} + v_{i}$ where $u_{i}$ and $v_{i}$ were employed to capture spatially correlated and unstructured extra variation in malaria distribution. Including both structured and unstructured random effects in a spatial analysis was crucial due to the lack of strong prior knowledge and the potential for unobserved confounders to manifest in various forms. The uncorrelated random effect was modelled by the zero-mean Gaussian distribution whereas the spatial random effect was described by the intrinsic conditional autoregressive model (ICAR) proposed by Besag et al. [19]. That is, conditionally, $u_{i} |{\varvec{u}}_{ - i} \sim N\left( {\overline{u}_{{\Omega_{i} }} ,{{\sigma_{u}^{2} } \mathord{\left/ {\vphantom {{\sigma_{u}^{2} } {n_{{\delta_{i} }} }}} \right. \kern-0pt} {n_{{\delta_{i} }} }}} \right)$ where ${\varvec{u}}_{ - i}$ is the vector containing the neighbouring effect of all except the *i*th area. $\Omega_{i}$, $n_{{\delta_{i} }}$ and $\overline{u}_{{\tau ,\delta_{i} }}$ are a set of adjacent neighbors, cardinality and the mean of the neighborhood of the *i*th province respectively, and $\sigma_{u}^{2}$ is the spatial component variance.

To model temporal variation, a linearity constraint could be imposed on the differential temporal trend, nonetheless a dynamic nonparametric formulation might be a better option for the linear predictor as there was no prior specific information for the trend. Various forms of temporal priors are available; this study considered the set of non-parametric models proposed by Knorr-Held [20], which are widely used in space–time disease mapping. These models allow us to account for temporal trends and different scenarios of potential differences in trends. There are three common forms of temporal random effect. The first one is to model $\lambda_{t}$ using a Gaussian exchangeable prior as $\lambda_{t} \sim Gaussian(0,\sigma_{\lambda }^{2} )$. The other two are the random walk (RW) priors of order 1 (RW1) and 2 (RW2), which can be expressed as $\lambda_{t}^{RW1} |\lambda_{t - 1}^{RW1} \sim Gaussian(\lambda_{t - 1}^{RW1} ,\sigma_{\lambda }^{2} )$ for RW1 and $\lambda_{t}^{RW2} |\lambda_{t - 1}^{RW2} ,\lambda_{t - 2}^{RW2} \sim Gaussian(2\lambda_{t - 1}^{RW2} + \lambda_{t - 2}^{RW2} ,\sigma_{\lambda }^{2} )$ for RW2. $\sigma_{\lambda }^{2}$ is the variance of the temporal random effect. The description of $\theta_{it}$ depends on the spatial and temporal random effects assumed to interact in the model. There are different types of interactions proposed in Bayesian disease mapping literature [20]. However, this study utilized three commonly used forms of interaction [21].

For type I interaction ($\theta_{1,it}$), the random effect was assumed to be interaction between the non-spatial, $v_{i}$, and exchangeable Gaussian temporal, $\lambda_{t}$, terms. According to Knorr-Held notation [20], the structure matrix ${\varvec{R}}_{\theta }$ for the prior of $\theta_{it}$ can be expressed as the Kronecker product of the interacting random effects. For the first type of interaction, the structure matrix can be written as ${\varvec{R}}_{{\theta_{1} }} = {\varvec{R}}_{v} \otimes {\varvec{R}}_{\lambda } = {\varvec{I}} \otimes \user2{I = I}$ since both $v_{i}$ and $\lambda_{t}$ do not have a specific spatiotemporal structure. Note that ***I*** here is the identity matrix. For the type II interaction ($\theta_{2,it}$), the interaction term combines the non-spatial with structured temporal random effects. Then the structure matrix can be described as ${\varvec{R}}_{{\theta_{2} }} = {\varvec{R}}_{v} \otimes {\varvec{R}}_{\lambda (RW)}$ where ${\varvec{R}}_{v} = {\varvec{I}}$ and ${\varvec{R}}_{\lambda (RW)}$ is based the neighbourhood structure specified by the order of random walk model. So $\theta_{2,it}$ can be formulated from the assumption of an autoregressive structure on the time component, which is independent from the ones of the other locations. The matrix ${\varvec{R}}_{{\theta_{2} }}$ then has a rank of *I*(*T* − 1) for a first-order and *I*(*T* − 2) for a second-order random walk model. For the last type of interaction,$\theta_{3,it}$ combines the unstructured temporal effect $\lambda_{t}$ and the spatially structured effect $u_{i}$. The structure matrix hence can be written as ${\varvec{R}}_{{\theta_{3} }} = {\varvec{R}}_{u} \otimes {\varvec{R}}_{\lambda }$ where ${\varvec{R}}_{u} = {\varvec{I}}$ and ${\varvec{R}}_{\lambda }$ is described through the intrinsic conditional autoregressive model. This results in the interaction with a spatial structure independent from the other time points and the structure matrix ${\varvec{R}}_{{\theta_{3} }}$ has a rank of *T*(*I* − 1). The precision parameters, which represent the reciprocal of variance, were implemented using a Log-Gamma distribution. Specifically, hyperparameters of 1 and 0.0005 were used for the CAR model, while hyperparameters of 1 and 0.00005 were used for the uncorrelated and random walk random effects. More details of model specifications of malaria incidence can be found in the supplementary document S1.

### Bayesian model selection procedure

In general, the primary goal of model selection is to choose the simplest model that provides the best fit to the observed data. In the context of hierarchical modeling discussed previously, several decisions need to be made regarding the inclusion of various fixed and random effects in the model. There are also many possible choices of space–time random effects. All of these considerations have an impact on both the mean estimated incidence and the association with potential risk factors. The process of choosing a model for a given set of spatiotemporal health data requires a series of model-fitting steps and investigations, and selection of appropriate mean and random effect structures for the observed data. However, model building typically should involve a balance of statistical and epidemiological considerations to effectively translate the findings to inform practical plans such as those for disease control and elimination.

An issue with spatiotemporal hierarchical modelling is that the model comprises two components: a fixed effect (the explanatory variables) and the random effects. Therefore, it is necessary to select not only the best explanatory variables but also an optimal random effects structure. In most cases, the focus is on the fixed effects. However, if the random effects are poorly chosen, this can affect the values and quality of the fixed effects because the random effects affect the standard errors of the slopes for the fixed effects. On the other hand, variation in the response variable that is not modeled in terms of fixed effects ends up in the random effects. Proposed strategies are available to work through the model selection process [22]; in this work, a top-down approach was employed to determine the optimal spatiotemporal mixed structure for malaria modelling. The following outlines the broad procedure of our two-step spatiotemporal model selection process.

The modeling process began with a comprehensive model that included all available explanatory variables, termed the optimal mean model. In cases where challenges such as a large number of explanatory variables or numerical issues arise, it is advisable to focus on variables most relevant to the research objective. For this study, all relevant climatic variables were included in the initial model. In the first step, the covariate model was used to determine the most suitable structure for the random component. Given that the fixed component already encompassed all pertinent explanatory variables, the random component was not expected to overlap with these variables. Multiple evaluation metrics were applied to compare different random effect specifications. In the second step, the optimal random structure identified in the first step was used to refit the data, employing various sets of explanatory variables. The best set of covariates was then selected based on various evaluation criteria, given the previously determined optimal random structure. Detailed comparisons and evaluation metrics are provided in the supplementary files S1-2.

To assess the relationship between malaria incidence and weather at the provincial level, the coefficient estimate for each climatic factor in the model was examined. This parameter reflects the strength of the association and can be interpreted epidemiologically as an incidence ratio. However, because the coefficient estimate represents a single point estimate, uncertainty quantification was also considered using exceedance probability. Within the Bayesian framework, exceedance probability is defined as the probability of the coefficient being greater than zero, serving as a Bayesian equivalent to the Frequentist p-value. A coefficient was deemed ‘significant’ when its exceedance probability exceeded the pre-specified significance level of 0.05.

## Results

**Fig. 2:**
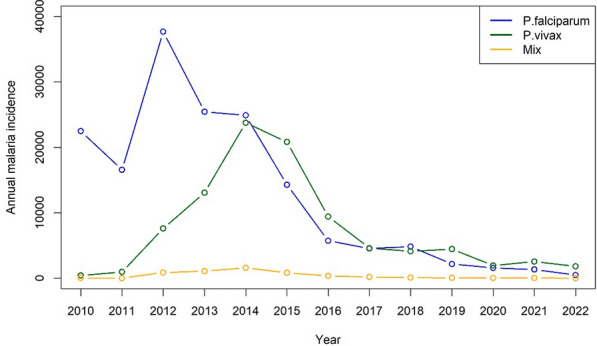
Plot of annual malaria incidence in Lao PDR during 2010–2022. The black line represents *P. falciparum*, the blue line *P. vivax* and the orange line shows mixed species infections

During the study period, a total of 161,947 cases of *P. falciparum*, 95,470 cases of *P. vivax*, and 5212 cases with mixed infection of *Plasmodium* species were reported. The proportion of *P. falciparum* to *P. vivax* cases decreased from 94.43% (16,553 cases) in 2010 to 26.43% (480 cases) in 2022. The annual parasite incidence (API) of *P. falciparum* declined from 351.98 cases per 10,000 in 2010 to 6.46 per 10,000 populations in 2022. However, the API of *P. vivax* increased over the same time period, from 1.86 to 24.44 per 10,000 between 2010 and 2022 (see Table 1). The incidence of *P. falciparum* peaked in 2012 and has been decreasing since, while the incidence of *P. vivax* and other species gradually increased during 2010–2012 and peaked in 2014, followed by a decline until 2022 (see Fig. 2).

**Table 1:** Malaria cases of *Plasmodium* species in Lao PDR during 2010–2022

Year	Population	*P. falciparum*			*P.vivax*			Mixed species		
		Cases	Percentage	API	Cases	Percentage	API	Cases	Percentage	API
2010	6,385,000	22,474	98.04	351.98	426	1.86	6.67	24	0.10	0.38
2011	6,514,000	16,552	94.51	254.10	962	5.49	14.77	0	0.00	0.00
2012	6,644,000	37,664	81.65	566.89	7,594	16.46	114.30	873	1.89	13.14
2013	6,809,000	25,436	64.27	373.56	13,064	33.01	191.86	1,079	2.73	15.85
2014	6,492,000	24,889	49.55	383.38	23,752	47.28	365.87	1,593	3.17	24.54
2015	6,787,000	14,261	39.74	210.12	20,805	57.97	306.54	822	2.29	12.11
2016	6,901,000	5,725	36.94	82.96	9,413	60.74	136.40	359	2.32	5.20
2017	7,013,000	4,548	48.76	64.85	4,592	49.23	65.48	188	2.02	2.68
2018	7,123,000	4,828	53.38	67.78	4,105	45.38	57.63	112	1.24	1.57
2019	7,231,000	2,168	32.38	29.98	4,448	66.44	61.51	79	1.18	1.09
2020	7,319,000	1,577	44.41	21.55	1,936	54.52	26.45	38	1.07	0.52
2021	7,425,000	1,345	34.26	18.11	2,557	65.13	34.44	24	0.61	0.32
2022	7,430,000	480	20.72	6.46	1,816	78.38	24.44	21	0.91	0.28

A general spatial pattern of high risk for *P. falciparum* was observed throughout the country in 2010 (see Fig. 1). Since then, the incidence has declined in the north, while most of the *P. falciparum* cases have been clustered in the south. For *P. vivax*, there were scattered cases in 2010 followed by an increase during 2012–2014, with the majority of cases in the south and a subsequent decline towards 2018. For mixed *Plasmodium* species, there were small numbers of cases with in 2010, and an increase in the south during 2012–2014. Cases with other species have continued to be reported in the south since then, with a slowly decreasing trend. It should be noted that due to limited resources for malaria diagnosis in the past, there might be overlapping numbers reported for both single-species and mixed infections. However, it is important to highlight that these cases with overlapping numbers were minimal and should not have a significant impact on the analysis.

Various spatiotemporal model formulations were constructed to assess the association of malaria incidence with climatic factors (see the supplementary file for more details) with evaluation measures and focused only on *P. falciparum* and *P. vivax* because those were the major public health interest of the Lao government. Tables 2, 3 show the various forms of model specification investigated in this study. Initially, random effect selection was performed, incorporating an optimal mean function with all climatic factors. Fifteen space–time random effect specifications were chosen to fit the provincial malaria incidence for both *P. falciparum* and *P. vivax*. Models 9 and 10 similarly had the best performance for both species; however, model 10 with spatial random effect had slightly better goodness of fit values. Consequently, the fixed effect selection was conducted using the random effects specified in model 10, which included BYM, non-parametric temporal trend, and type 1 interaction terms.

**Table 2:** Model evaluation measures of random and fixed effect selection for *P. falciparum* malaria model

Procedure	Model	Specification	DIC^c^	WAIC^c^	CPO^c^	MLIK^c^	Bias	RMSE^c^	Correlation
Random effect	1	$\beta_{0} + v_{i}$	181,406.9	67,194.17	Inf	− 90,903.3	− 0.0106	131.6307	0.7903
Selection	2	$\beta_{0} + v_{i} + u_{i}$	181,407.3	67,194.3	Inf	− 90,893.3	− 0.0106	131.6307	0.7903
	3	$\beta_{0} + v_{i} + \lambda_{t}$	46,205.3	48,208.99	25,037.48	− 23,529.4	− 0.0325	67.6067	0.8760
	4	$\beta_{0} + v_{i} + u_{i} + \lambda_{t}$	46,205.59	48,208.54	25,559.38	− 23,518.7	− 0.0325	67.6065	0.8760
	5	$\beta_{0} + v_{i} + \lambda_{t}^{RW1}$	46,200.73	48,075.19	26,022.67	− 23,362.8	− 0.0106	131.6307	0.7903
	6	$\beta_{0} + v_{i} + u_{i} + \lambda_{t}^{RW1}$	48,157.07	49,256.44	23,691.07	− 24,348.5	− 0.0301	70.0308	0.8747
	7	$\beta_{0} + v_{i} + \lambda_{t}^{RW2}$	46,194.91	47,907.44	25,046.94	− 23,386.9	− 0.0298	67.7094	0.8762
	8	$\beta_{0} + v_{i} + u_{i} + \lambda_{t}^{RW2}$	46,195.34	47,907.74	25,094.78	− 23,375.8	− 0.0298	67.7095	0.8762
	9	$\beta_{0} + v_{i} + \lambda_{t}^{{}} + \theta_{1,it}$	10,985.4	10,911.64	33,316.18	− 7236.58	− 0.2672	0.7904	0.9327
	10	$\beta_{0} + v_{i} + u_{i} + \lambda_{t}^{{}} + \theta_{1,it}$	10,973.87	10,891.31	31,447.2	− 7209.36	− 0.2685	0.7639	0.9332
	11	$\beta_{0} + v_{i} + \lambda_{t}^{RW1} + \theta_{2,it}$	14,672.91	15,203.41	8763.795	− 32,472.8	− 1.4479	2.8940	0.9171
	12	$\beta_{0} + v_{i} + u_{i} + \lambda_{t}^{RW1} + \theta_{2,it}$	14,674.3	15,202.31	8831.923	− 32,459.7	− 1.4511	2.8146	0.9177
	13	$\beta_{0} + v_{i} + \lambda_{t}^{RW2} + \theta_{2,it}$	14,826.3	15,844.95	9040.776	− 40,869.9	− 1.4314	5.1483	0.9154
	14	$\beta_{0} + v_{i} + u_{i} + \lambda_{t}^{RW2} + \theta_{1,it}$	14,939.8	15,944.28	9128.928	− 41,275.1	− 1.4602	5.1205	0.9136
	15	$\beta_{0} + u_{i} + v_{i} + \lambda_{t}^{{}} + \theta_{3,it}$	15,436.49	16,617.31	23,559.29	− 29,405.3	− 1.5130	2.1125	0.9247
Fixed effect^a,b^	1	Rain+maxt+maxh+mint + minh	10,973.13	1512.876	10,889.99	1023.932	31,598.42	− 7201.91	− 0.2787
Selection	2	Rain+mint+minh	10,979.5	1518.017	10,900.66	1024.109	31,651.24	− 7204.51	− 0.2678
	3	Maxt+maxh+mint+minh	11,002.67	1532.58	10,926.89	1040.913	33,108.23	− 7226.83	− 0.2721
	4	Mint+minh	10,989.17	1512.258	10,908.24	1025.569	31,871.88	− 7202.71	− 0.2684
	5	Rain+maxt+maxh+mint+minh	10,982.97	1517.275	10,908.4	1032.417	35,325.98	− 7202.67	− 0.2694
	6	Rain+mint+minh	10,976.79	1507.717	10,898.3	1024.181	35,201.25	− 7201.7	− 0.2678
	7	Maxt+maxh+mint+minh	10,974.58	1498.758	10,893.31	1017.459	32,288.15	− 7161.78	− 0.2660
	8	Mint + minh	10,987.21	1510.388	10,905.75	1024.075	31,963.69	− 7185.63	− 0.2682

^a^ *min.h, max.h*  minimum and maximum humidity, *min.t, max.t*  minimum and maximum temperature, *rain*  rainfall, *sun*  sunshine

^b^Models 1–6 = non-spatial random slope, models 7–12 = spatial random slope

^c^*DIC*  Deviance information criterion, *WAIC* = Widely applicable Bayesian information criterion, *CPO* Conditional predictive ordinate, *MLIK* Marginal likelihood, *RMSE* Root mean squared error

**Table 3:** Model evaluation measures of random and fixed effect selection for *P. vivax* malaria model

Procedure	Model	Specification	DIC^c^	WAIC^c^	CPO^c^	MLIK^c^	Bias	RMSE^c^	Correlation
Random effect	1	$\beta_{0} + v_{i}$	119,186.8	56,634.81	Inf	− 59,896.2	− 0.0106	89.0503	0.7358
selection	2	$\beta_{0} + v_{i} + u_{i}$	119,186.7	56,633.71	Inf	− 59,885.5	− 0.0106	89.0503	0.7358
	3	$\beta_{0} + v_{i} + \lambda_{t}$	36,065.61	38,347.51	20,456.28	− 18,596	− 0.0320	32.7608	0.8427
	4	$\beta_{0} + v_{i} + u_{i} + \lambda_{t}$	36,063.63	38,340.74	20,585.4	− 18,600.6	− 0.0320	32.7608	0.8427
	5	$\beta_{0} + v_{i} + \lambda_{t}^{RW1}$	36,052.27	38,116.93	20,536.08	− 18,410.4	− 0.0106	89.0503	0.7358
	6	$\beta_{0} + v_{i} + u_{i} + \lambda_{t}^{RW1}$	36,602.6	38,382.69	20,760.53	− 18,629.4	− 0.0283	33.0353	0.8394
	7	$\beta_{0} + v_{i} + \lambda_{t}^{RW2}$	36,055.87	37,887.29	20,406.82	− 18,441.8	− 0.0270	32.8099	0.8426
	8	$\beta_{0} + v_{i} + u_{i} + \lambda_{t}^{RW2}$	36,056.13	37,886.59	20,406.68	− 18,431.6	− 0.0270	32.8100	0.8425
	9	$\beta_{0} + v_{i} + \lambda_{t}^{{}} + \theta_{1,it}$	11,385.99	11,523.47	40,090.94	− 7405.86	− 0.3141	0.6947	0.9395
	10	$\beta_{0} + v_{i} + u_{i} + \lambda_{t}^{{}} + \theta_{1,it}$	11,381.44	11,516.81	40,250.92	− 7387.55	− 0.3133	0.6976	0.9398
	11	$\beta_{0} + v_{i} + \lambda_{t}^{RW1} + \theta_{2,it}$	14,454	14,950.91	8636.865	− 32,427.8	− 1.4559	2.6245	0.9210
	12	$\beta_{0} + v_{i} + u_{i} + \lambda_{t}^{RW1} + \theta_{2,it}$	14,454.29	14,950.89	8632.332	− 32,417.5	− 1.4557	2.6267	0.9210
	13	$\beta_{0} + v_{i} + \lambda_{t}^{RW2} + \theta_{2,it}$	14,576.72	15,452.84	8550.66	− 40,701.1	− 1.4190	4.5609	0.9154
	14	$\beta_{0} + v_{i} + u_{i} + \lambda_{t}^{RW2} + \theta_{1,it}$	14,694.53	15,573.88	8629.205	− 41,118.9	− 1.4477	4.6046	0.9130
	15	$\beta_{0} + u_{i} + v_{i} + \lambda_{t}^{{}} + \theta_{3,it}$	15,539.09	17,154.28	33,496.9	− 29,717.8	− 1.5544	2.1105	0.9104
Fixed effect^a,b^	1	Rain+max t+max h+min t+min h	11,374.72	11,428.57	40,116.61	− 7388.92	− 0.3152	0.6770	0.9498
selection	2	Rain+min t + min h	11,390.65	11,534.78	40,136.86	− 7384.52	− 0.3144	0.6927	0.9496
	3	Maxt+max h + min t+min h	11,397.56	11,540.48	39,980.59	− 7385.89	− 0.3157	0.6896	0.9401
	4	Min t+min h	11,408.63	11,563.48	40,392.35	− 7386.67	− 0.3168	0.6863	0.9403
	5	Rain+max t+max h+min t+min h	11,379.35	11,505.08	39,767.44	− 7354.68	− 0.3110	0.7115	0.9386
	6	Rain + min t+min h	11,388.55	11,531.03	39,718.77	− 7385.94	− 0.3136	0.6981	0.9393
	7	Max t+max h+min t+min h	11,391.3	11,533.61	40,067.34	− 7335.44	− 0.3155	0.6889	0.9401
	8	Min t+min h	11,407.41	11,561.87	40,000.31	− 7373.49	− 0.3168	0.6857	0.9402

^a^ *min.h, max.h*  minimum and maximum humidity, *min.t, max.t*  minimum and maximum temperature, *rain*  rainfall, *sun*  sunshine

^b^Models 1–6 = non-spatial random slope; models 7–12 = spatial random slope

^c^*DIC*  Deviance information criterion, *WAIC*  Widely applicable Bayesian information criterion, *CPO* Conditional predictive ordinate, *MLIK* Marginal likelihood, *RMSE* Root mean squared error

For fixed effect selection, six combinations of climatic factors were examined, as detailed in models 1–6, with a non-spatial random slope applied to each climatic coefficient. However, we also modeled the spatial random slope for the same covariates in models 7–12 to examine the spatial effects of climatic associations at the provincial level. The full mean model with all climatic variables had the best performance for both spatial and non-spatial random coefficients for both *P. falciparum* and *P. vivax*; the non-spatial random slope models were slightly better in terms of goodness of fit measures. Thus, with the 2-step model selection procedure, the overall optimal model for both species was model 1 (as in fixed effect selection) with all climatic factors combined with BYM, non-parametric temporal trend and type1 interaction random effect terms (as model 10 in random effect selection).

**Fig. 3:**
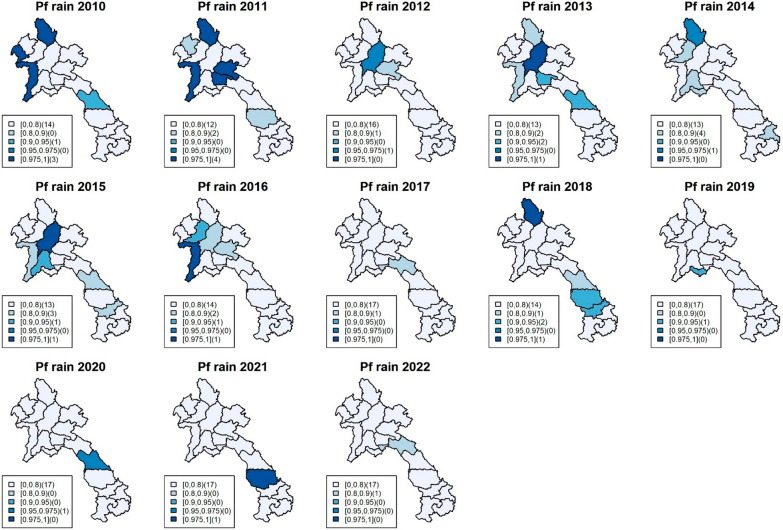
Maps of exceedance probability of the provincial association between *P. falciparum* incidence and rainfall in Lao PDR during the study period

**Fig. 4:**
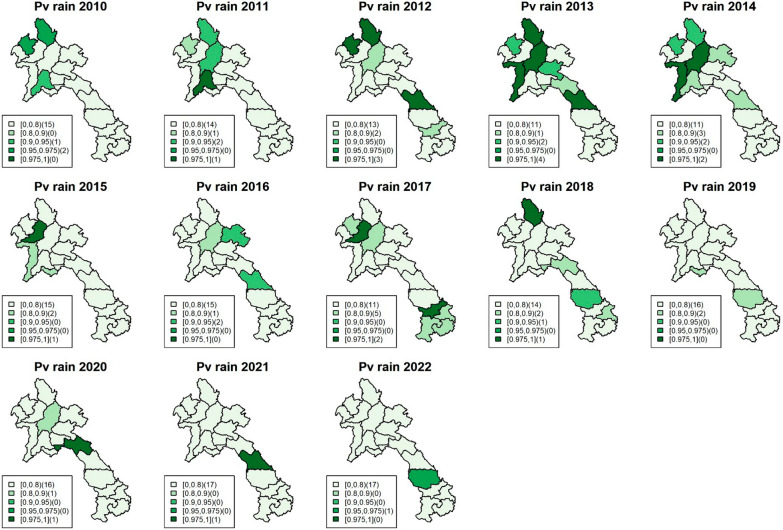
Maps of exceedance probability of the provincial association between *P. vivax* incidence and rainfall in Lao PDR during the study period

**Fig. 5:**
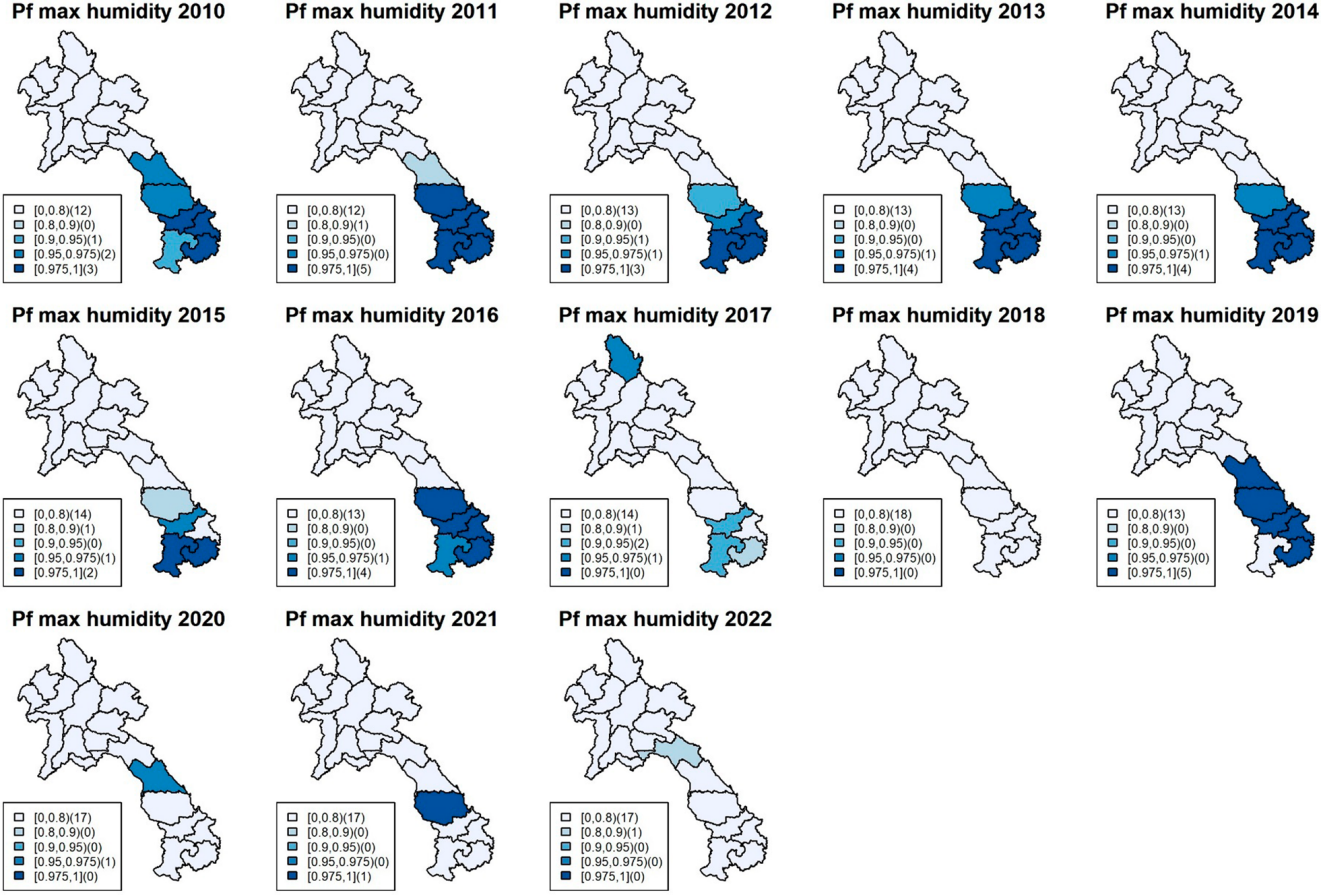
Maps of exceedance probability of the provincial association between *P. falciparum* incidence and maximum humidity in Lao PDR during the study period

**Fig. 6:**
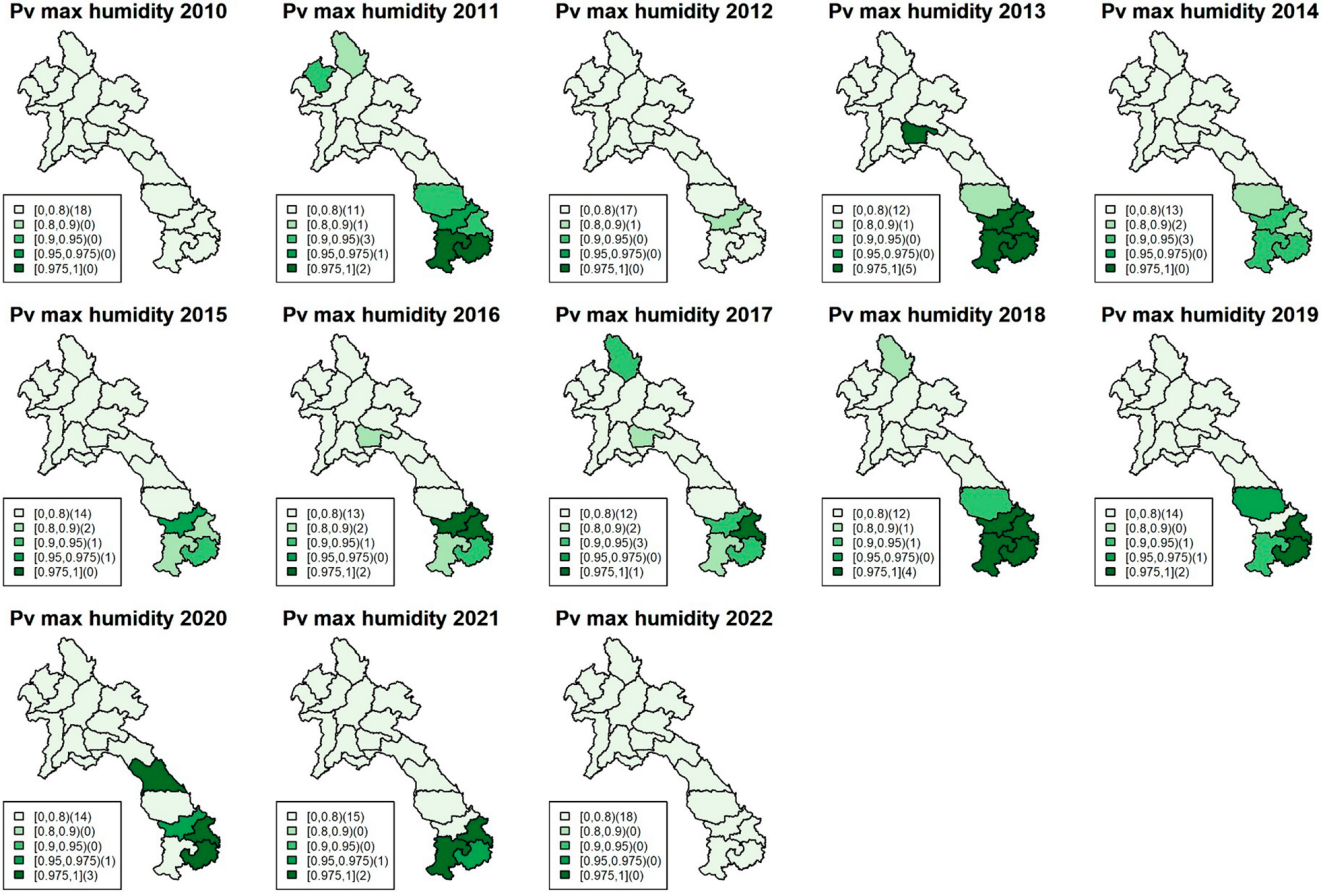
Maps of exceedance probability of the provincial association between *P. vivax* incidence and maximum humidity in Lao PDR during the study period

The model incorporated several climatic factors, but the focus here was on the most significant variables: rainfall and maximum humidity (Figs. 3, 4, 5 and 6), while other results can be found in the supplementary documents S3-S5, which include exceedance probability maps and tables of coefficient estimates. All climatic variables were included in the model, and the incidence rate ratio (IRR) for each factor was adjusted for the others. Figures 3 and 4 show the exceedance probability of the provincial association between malaria incidence and rainfall, while Figs. 5 and 6 display the exceedance probability of the provincial association with maximum humidity during the study period in Lao PDR. The estimated IRRs are provided in Tables S4-S5 in the supplementary file.

As mentioned, the climatic variables most strongly associated with incidence during the study period were rainfall and maximum humidity. Rainfall (monthly average in millimetres) was significant in the north and central regions during 2010–2011, with average falciparum IRRs ranging from 1.0046 (95%CI 1.001–1.008) to 1.0057 (95%CI 1.002–1.011), while maximum humidity (monthly average in percentage) was persistently significant in the south, with IRRs ranging from 1.024 (95%CI 1.007–1.041) to 1.12 (95%CI 1.076–1.177). For vivax incidence, the climatic association was also significant. Rainfall was particularly associated with the outbreak during 2012–2015 in the north and central regions, with IRRs ranging from 1.0023 (95%CI 1.001–1.0048) to 1.0065 (95%CI 1.0022–1.012), while maximum humidity was persistently associated with vivax incidence, particularly in the south, with IRRs ranging from 1.0191 (95%CI 1.0017–1.0362) to 1.158 (95% CI 1.076–1.239).

**Fig. 7:**
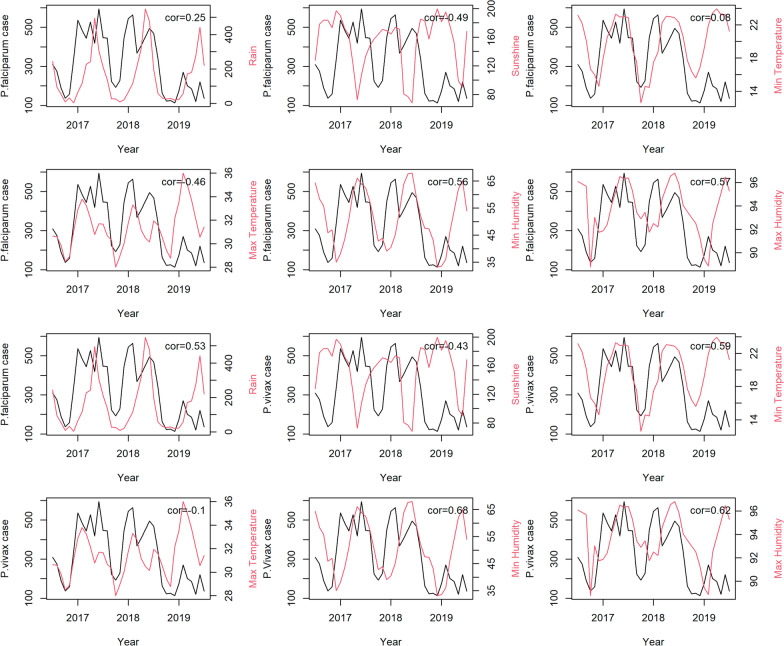
Plots of monthly malaria incidence (black) with the Spearman’s association for each climatic variable (red) in Lao PDR during 2017–2019

The study findings revealed that rainfall and maximum humidity were the most influential climatic variables associated with malaria incidence during the study period. For falciparum malaria, an increase of 1 mm of rainfall was found to be associated with an average increase of 0.5% in malaria cases among the provincial population in the north and central regions. Similarly, a 1% increase in relative humidity was linked to a rise of about 10% in falciparum malaria cases in the southern region. Regarding vivax malaria, increased rainfall during the period of 2012–2015 in the north and central regions was associated with a higher risk of vivax malaria, ranging from approximately 0.2% to 0.65% per 1 mm of rainfall. Furthermore, higher levels of maximum humidity consistently showed a significant association with a higher risk of vivax malaria in the south, with an increase ranging from approximately 2% to 16% on average. These associations were assumed to approximately exhibit linearity within the range of climate data during the study period, as detailed in supplementary document S6. In addition, the figures show heterogeneity of spatiotemporal association with climatic factors across the country, with stronger associations found in the south, where malaria incidence has been higher for both falciparum and vivax. Figure 7 shows line plots of each climatic variable with monthly malaria incidence and their Spearman’s association estimates, suggesting a relationship between provincial malaria incidence and seasonality of climatic factors.

## Discussion

Climatic data are frequently used to model spatiotemporal variation in malaria

Transmission. For Lao’s national malaria control programme, a 10-year surveillance dataset (2011–2020) was employed to explore the meteorological associations with malaria incidence in the country. The present study has demonstrated changes of *P. falciparum* and *P. vivax* malaria incidence in Lao PDR. It was observed that malaria transmission has generally declined in the northern and central regions, though high-risk areas persist in the south. As countries in the GMS move towards malaria elimination, *P. falciparum* incidence declines more rapidly than the incidence of *P. vivax*, as in Lao PDR, in part because of the greater effectiveness of vector control interventions [23]. In contrast, treating all stages of the parasite (radical cure) is a critical strategy for the successful control and ultimate elimination of *P. vivax*. Malaria transmission continues to decline in Lao PDR, and incidence is becoming increasingly heterogeneous, with most cases now concentrated in a relatively few provinces, particularly in the south [24]. Such patterns are consistent with those of other countries in the region. Therefore, spatially targeting interventions and associated resources are likely to achieve better results than a uniform approach to the distribution and delivery of malaria reduction interventions [25, 26].

This study applied various Bayesian spatiotemporal models to retrospectively analyse observed malaria surveillance data and estimate the climatic associations that can be useful for appropriate disease control and elimination planning. However, constructing space–time models presents a challenge, as they contain both fixed effects (climatic variables) and random effects. To address this challenge, a two-step Bayesian model selection framework was proposed to identify the optimal model for the investigation. Nonetheless, building an appropriate spatiotemporal model for a given set of epidemiological data is an iterative process that involves a series of model-fitting steps and investigations, and the selection of appropriate fixed and random effect structures for the observed data. Model building typically requires a balance of statistical and subject matter considerations, and there is no single strategy that applies to every application. While the model-building procedure, proposed in this project serves as an example and not a hard-and-fast rule for space–time model selection, it offers a flexible modelling approach that can accommodate and potentially select between a wide range of space–time linear predictors. This may be useful in studying spatiotemporal health outcomes in different settings.

In addition to applying space–time random effects to account for spatiotemporal correlation, which have been utilized in malaria mapping, particularly with Gaussian and autoregressive models for spatial and temporal effects [11], this study also integrated additional priors to enable a more thorough comparison. Sensitivity analyses were conducted to evaluate the robustness of the findings by varying model assumptions, including priors, distributions, and the structural components of random effects. The consistency of results across the best models, as assessed by various evaluation metrics, indicates robustness, whereas any discrepancies highlight the potential impact of specific assumptions on the results. A notable enhancement in model performance was achieved by incorporating a space–time interaction term, which is recognized for improving model precision [27]. By integrating these interaction terms, these models capture nuanced variations in malaria incidence across regions and over time. This precision enables health authorities to pinpoint specific associations and periods of heightened transmission, facilitating targeted and timely interventions.

Several criteria were employed to investigate the association with climate. DIC can assess overall model fit in Bayesian frameworks but can be sensitive to model complexity, potentially leading to overfitting while WAIC, an improved fully Bayesian alternative, uses the entire posterior distribution, making it robust but computationally intensive. CPO evaluates model fit through cross-validation by providing posterior probabilities for observations, though it is computationally demanding with large datasets. MLIK measures the probability of observed data under a model but does not directly account for model complexity. Bias measures systematic error, while RMSE combines bias and variance, providing a comprehensive measure of estimation accuracy, though it is sensitive to outliers. Spearman’s correlation coefficient assesses rank correlation between observed and estimated values, suitable for non-normally distributed data but not capturing the magnitude of differences. Information criteria such as DIC and WAIC have been used as primary criteria to compare modeling options in spatiotemporal Bayesian disease mapping and have been validated to select the true model specification in simulation studies [28, 29].

When investigating the association between climatic variables and malaria incidence, it is important to consider the biology of the transmission process. This is because changes in explanatory factors may not immediately translate to changes in malaria transmission, partly due to vector biology, which can result in a time lag in the relationship. Therefore, it is important to account for this lag effect in the analyses. Although the malaria incidence data were collected on a monthly basis, the possibility of lag effects was considered, with the expectation that any such effects would be minimal in the dataset. Nonetheless, distributed lag nonlinear models have been shown to be an effective tool for accounting for lags when data are available in a finer temporal scale (see examples [2, 3, 30–32]).

The results of this study demonstrate that rainfall and humidity are significant drivers of spatiotemporal patterns of malaria incidence in Lao PDR, which is consistent with similar findings in other studies conducted in the GMS region [33, 34]. The indirect effect of relative humidity on both the development of parasites and the activity and survival of anopheline mosquitoes has been previously reported [35], with low humidity limiting the distribution and abundance of mosquito vectors in China [36]. An association between relative humidity and *P. falciparum* was not found in this subtropical area, which is consistent with a previous study carried out in a tropical rain forest area along the China–Laos border, in southern Yunnan [37].

In addition, other environmental and ecological factors significantly influence malaria transmission. Among these, rainfall and temperature are particularly important. Heavy rainfall can wash away mosquito breeding sites, while temperature affects both mosquito larval development and parasite maturation within vectors [38, 39]. Precipitation, which is directly related to rainfall, significantly impacts the bionomics of mosquito vectors [40]. Environmental modifications, such as dam construction and irrigation projects, can also alter the distribution of mosquito breeding sites [41]. Therefore, understanding climatic influences is crucial for effective malaria control. The results provide critical insights for developing targeted local malaria surveillance-response systems and implementing timely intervention initiatives. By incorporating climate investigations into public health strategies, authorities can better respond to malaria outbreaks, thereby enhancing control efforts and advancing toward malaria elimination goals.

Recent studies have emphasized the need for interdisciplinary approaches to develop an early warning system that incorporates climate information to evaluate the implications of weather variability on malaria transmission [42, 43]. A better understanding of the link between malaria incidence and interannual climate variability, particularly in regions with high transmission rates, could facilitate the development of robust malaria surveillance systems and strengthen planning and actionable disease control strategies by public health authorities in the region. The findings of this study indicate significant statistical associations between climatic factors and malaria incidence, underscoring the practical potential of weather information in climate-driven malaria surveillance and control efforts, particularly in high-risk areas. While spatiotemporal random effects partially address unmeasured variables, integrating data on interventions can enhance the ability to distinguish climate effects from intervention impacts. This enhanced understanding of intervention impacts could also optimize resource allocation for more effective malaria prevention and control initiatives. Furthermore, integrating climate data into malaria prevention strategies can enhance adaptive management approaches. By anticipating seasonal variations in transmission risk based on climate forecasts, public health officials can pre-position medical supplies and deploy healthcare and vector control personnel to respond to potential outbreaks. This proactive approach can mitigate the burden of malaria on healthcare systems and reduce morbidity and mortality associated with the disease.

The limitations of the study should also be acknowledged. Investigating the space–time pattern of malaria transmission at a provincial level may mask local underlying patterns of disease through averaging [44]. Therefore, using a finer geographic scale such as a district or subdistrict may provide a more detailed view of important local variations in malaria transmission and could guide malaria control efforts and resource allocation, particularly in areas where transmission is decreasing towards elimination. Another limitation of this study is the assumption of a linear relationship between malaria incidence and weather variables, whereas this association might be nonlinear. Nevertheless, the inclusion of flexible space–time random effects helps in capturing potential spatiotemporal nonlinear variations. Furthermore, the precise pattern of this relationship has not been extensively examined. Thus, while approximating a linear association with the integration of space–time random terms seems reasonable, future research could further explore more modelling techniques to address these potential nonlinearities. Understanding these non-linear relationships can enhance comprehension of the complex interactions between climate and malaria transmission. This knowledge is crucial for developing adaptive management strategies that effectively respond to changing environmental conditions and evolving epidemiological trends. Incorporating non-linearities into models can improve the predictive accuracy by capturing these intricate relationships more precisely.

This improvement has significant practical implications for public health policy and malaria prevention strategies. Enhanced predictive models can inform the development of early warning systems that integrate climate variables, allowing for timely and targeted interventions. Public health authorities can use these models to anticipate periods of increased transmission risk and allocate resources more efficiently, such as pre-positioning medical supplies and deploying healthcare personnel to high-risk areas. Furthermore, these insights can guide the design of community-based prevention programmes, ensuring that they are tailored to local climatic conditions. This approach can improve the effectiveness of interventions such as insecticide-treated bed nets and indoor residual spraying.

It is also important to address additional explanatory variables to provide a comprehensive understanding of malaria in the country. This may include factors such as mosquito vector behaviour, community behaviour, access to and delivery of health services, and other eco-bio-social factors that affect malaria incidence. Despite these limitations, the proposed flexible spatiotemporal modelling framework with a two-step model selection approach can be easily applied when more resources and data become available. The investigation provides valuable insights into the spatiotemporal patterns of malaria incidence, which can aid decision-making in malaria control and elimination activities in Lao PDR.

## Conclusions

The goal of malaria elimination in Lao PDR by 2030 countrywide and by 2025 in the north is ambitious, but achievable with a well-informed and coordinated approach. As weather and environment play a critical role in malaria transmission, understanding the spatial and temporal dynamics of the climate-malaria relationship is essential for effective planning and implementation of control activities. The study considered several climatic factors, providing epidemiologically meaningful quantification that can inform policy-making by public health workers. The findings can be utilized to support the malaria surveillance system and optimize resource allocation towards achieving the goal of malaria elimination in Lao PDR.

To further advance towards malaria elimination, it may be important to implement robust malaria surveillance systems that integrate real-time climate data. This integration can enhance the ability to identify transmission hotspots and seasonal variations promptly, facilitating targeted response efforts to prevent outbreaks. Moreover, future research could explore various climatic variables, employ diverse model structures, and extend the application of these models to other infectious diseases. These activities can not only deepen the understanding of climate-disease interactions but also serve as a valuable reference for advancing knowledge and informing evidence-based policies in infectious disease control and prevention. By continuing with elimination activities and research efforts, progress towards malaria elimination in Lao PDR can significantly contribute to global health initiatives and improve the well-being of communities affected by malaria.

### Supplementary Information

Additional file 1.

## Data Availability

The data that support the findings of this study were obtained from Center of Malariology, Parasitology, and Entomology, Vientiane, Lao PDR, but restrictions apply to the availability of these data, which were used with permission for the current study, and are therefore not publicly available. No datasets were generated or analysed during the current study.

## Abbreviations

GMS: Greater Mekong Sub-region
API: Annual parasite incidence
CMPE: Center for Malaria Parasitology and Entomology of Lao PDR
DHIS2: District health information system 2
ICAR: Intrinsic conditional autoregressive model
RW: Random walk
CPO: Conditional predictive ordinate
MLIK: Marginal likelihood
DIC: Deviance information criterion
WAIC: Widely applicable Bayesian information criterion
INLA: Integrated nested Laplace approximation
RMSE: Root mean squared error
