# Supplementary material for: Spatiotemporal patterns and association with climate for malaria elimination in Lao PDR: a hierarchical modelling analysis with two-step Bayesian model selection
Source: Malar J. 2024 Aug 4;23:231. doi: 10.1186/s12936-024-05064-0 (PMC11298089; doi:10.1186/s12936-024-05064-0)
Supplement: Supplementary file 1 — Additional file 1. [file 12936_2024_5064_MOESM1_ESM.docx]

**Spatiotemporal patterns and association with climate for malaria elimination in Lao PDR: a hierarchical modeling analysis with two-step Bayesian model selection**

**S1: Spatiotemporal hierarchical modeling formulation**

In this study, we considered a range of spatiotemporal malaria incidence models, focusing on those that were most relevant for assessing the influence of climatic variables. Table S1 below provides an overview of all the model forms used. Models 1-2 included only spatially-varying random intercept terms, with the spatial random effect modeled using either an independent zero-mean Gaussian prior (model 1) or both the unstructured and Besag (BYM) prior (model 2) [1]. Models 3-8 included temporal terms with both Gaussian exchangeable and random walk priors. To capture extra space-time variation, we added an additional interaction between spatial and temporal terms in models 9-15. While there are many possible model specifications, we believe these models were the most sensible for our study's specific aims.

| Model | Random effect specification |
| --- | --- |
| 1 | $\beta_{0}+v_{i}$ |
| 2 | $\beta_{0}+v_{i}+u_{i}$ |
| 3 | $\beta_{0}+v_{i}+\lambda_{t}$ |
| 4 | $\beta_{0}+v_{i}+u_{i}+\lambda_{t}$ |
| 5 | $\beta_{0}+v_{i}+\lambda_{t}^{RW1}$ |
| 6 | $\beta_{0}+v_{i}+u_{i}+\lambda_{t}^{RW1}$ |
| 7 | $\beta_{0}+v_{i}+\lambda_{t}^{RW2}$ |
| 8 | $\beta_{0}+v_{i}+u_{i}+\lambda_{t}^{RW2}$ |
| 9 | $\beta_{0}+v_{i}+\lambda_{t}+\theta_{1,it}$ |
| 10 | $\beta_{0}+v_{i}+u_{i}+\lambda_{t}+\theta_{1,it}$ |
| 11 | $\beta_{0}+v_{i}+\lambda_{t}^{RW1}+\theta_{2,ti}$ |
| 12 | $\beta_{0}+v_{i}+u_{i}+\lambda_{t}^{RW1}+\theta_{2,it}$ |
| 13 | $\beta_{0}+v_{i}+\lambda_{t}^{RW2}+\theta_{2,it}$ |
| 14 | $\beta_{0}+v_{i}+u_{i}+\lambda_{t}^{RW2}+\theta_{1,it}$ |
| 15 | $\beta_{0}+u_{i}+v_{i}+\lambda_{t}+\theta_{3,it}$ |

**Table S1.** Model specifications with different forms of space-time random effects.

**S2: Model sensitivity analysis and selection criteria**

To evaluate the sensitivity and performance of our models, we employed a range of evaluation metrics to measure their outcomes. We used seven metrics, namely deviance information criterion (DIC), Watanabe-Akaike information criterion (WAIC), marginal likelihood (MLIK), conditional predictive ordinate (CPO), bias, root mean squared error (RMSE), and Spearman’s correlation coefficient. These metrics were chosen to assess different aspects of the models' performance.

The goodness-of-fit (GOF) measures were used to evaluate whether the data in space and time fit the model adequately. We used a set of common GOF measures, including the information criteria. The DIC [2] has been widely used as a measure of overall model fit in Bayesian settings, and it is a generalization of the Akaike information criterion (AIC) in the Frequentist framework. Another widely applicable information criterion is the WAIC [3], which can be viewed as an improved version of the DIC. Unlike DIC, WAIC is fully Bayesian and uses the entire posterior distribution. It is robust to different parametrizations and is also valid for singular models [4].

The next metric is the conditional predictive ordinate (CPO) [5]. This metric is a cross-validation criterion for model assessment that is computed for each observation as $CPO_{st}=P(y_{st}|y_{-st})$. Hence, for each observation the conditional predictive ordinate is the posterior probability of observing that observation when the model is fit using all data but the observation at location *s* and time *t*. Large values indicate a better fit of the model to the data, while small values indicate a bad fitting of the model. The conditional predictive ordinate measure for each model then can be summarized as $CPO=\sum_{t} \sum_{s} CPO_{st}$with bigger values pointing to a better model fit. Another goodness of fit measure used in valuation is the marginal likelihood of a model is the probability of the observed data under a given model, i.e., $P(y)$. When a set of $I$ models $\{M_{i}\}_{i=1}^{I}$ is considered, their respective marginal likelihoods can be represented as $P(y|M_{i})$ to indicate that they are different for different models.

The next measure we used was bias, which represents the average difference between the observed incidence and its estimate across locations and time periods in the study. A value close to zero is desirable for this metric. To assess the estimation uncertainty, we also calculated the root mean squared error (RMSE), which is the square root of the average squared deviation between the observed and estimated means across the provinces and study time periods. The RMSE considers both bias and variance of the estimates. We also computed the Spearman's correlation coefficient between the observed and estimated malaria cases. This measure is a nonparametric alternative to the Pearson product-moment correlation coefficient and assesses the strength and direction of association between two ranked variables. Given that the malaria incidence data in the elimination period are typically not normally distributed [6, 7], the Spearman’s correlation coefficient may be more suitable than the Pearson’s correlation coefficient in our investigation.

**S3: Maps of exceedance probability of the climatic association between *P. falciparum* (Pf) and *P. vivax* (Pv) malaria incidence and climatic factors during the study period in Lao PDR**


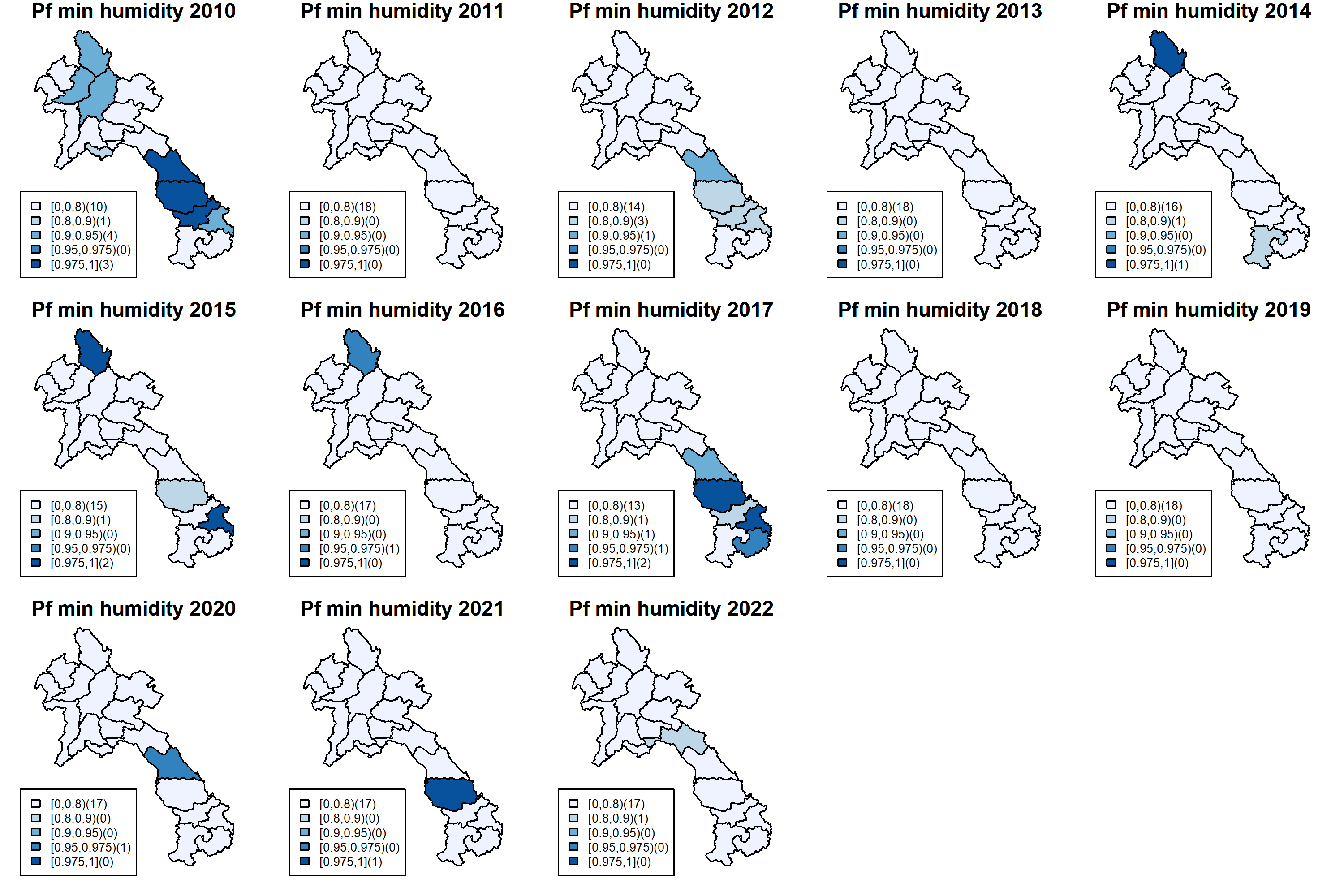


**Figure S3.1** Maps of exceedance probability of association between *P. falciparum* (Pf) incidence and minimum humidity in Lao PDR.


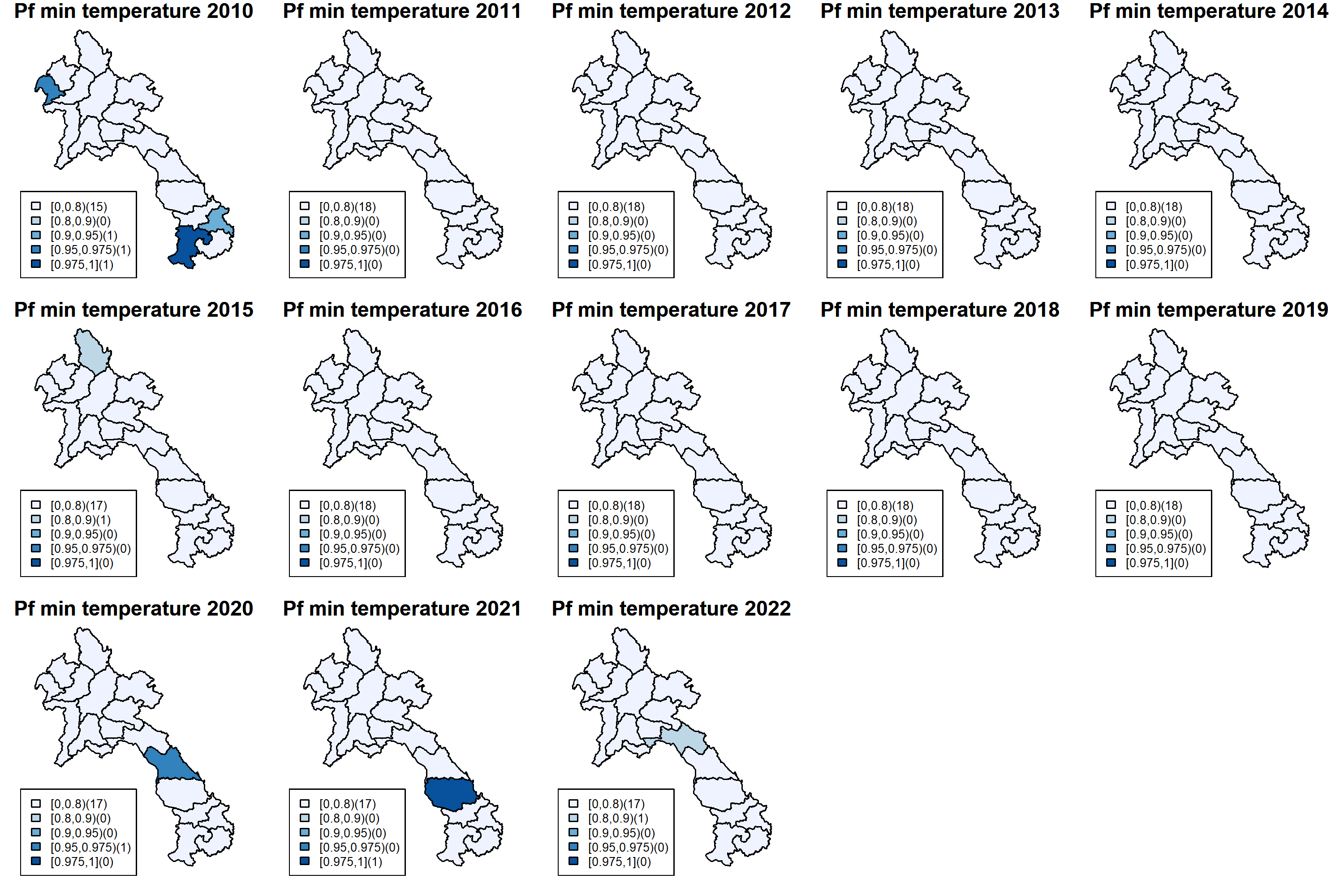


**Figure S3.2** Maps of exceedance probability of association between *P. falciparum* (Pf) incidence and minimum temperature in Lao PDR.


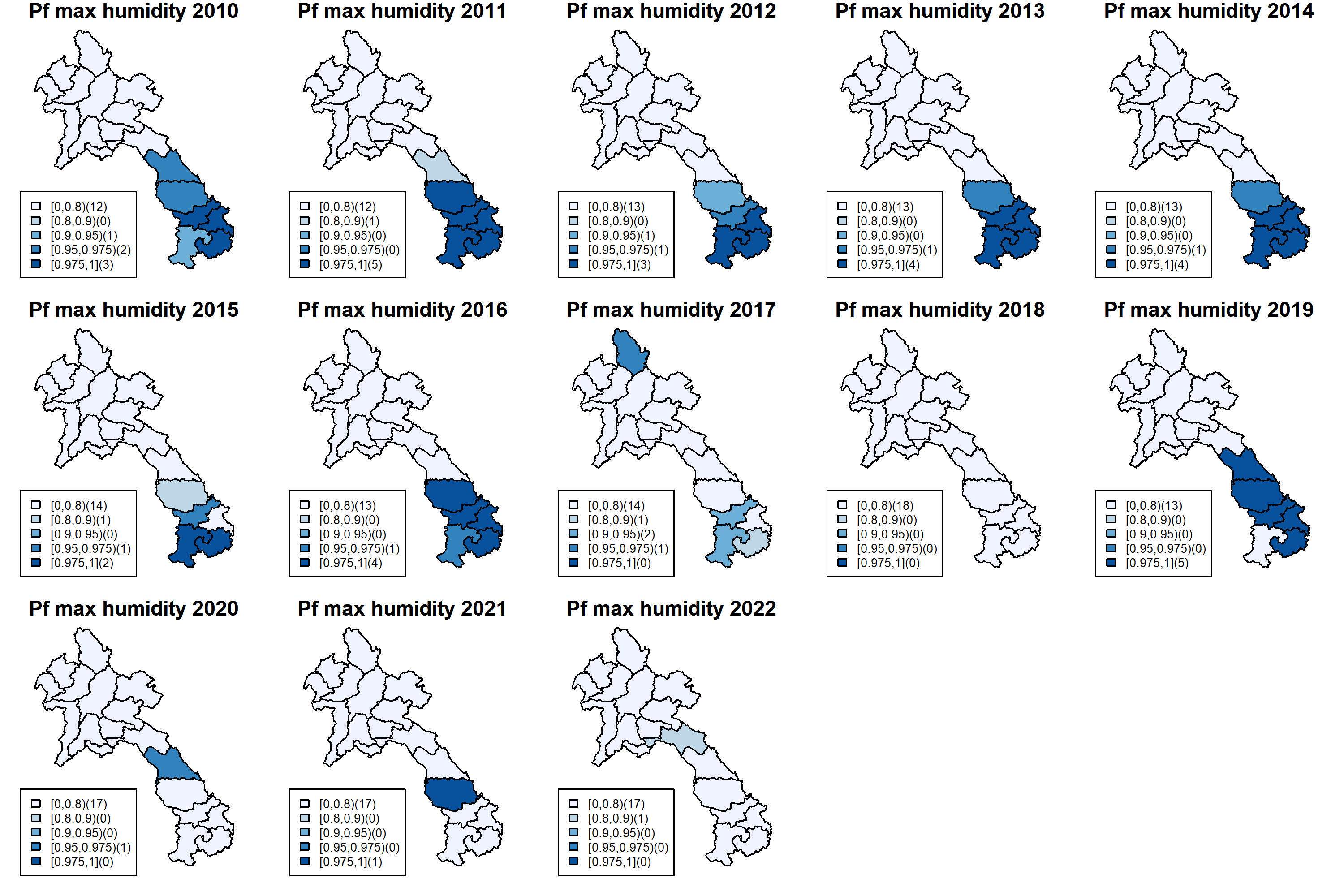


**Figure S3.3** Maps of exceedance probability of association between *P. falciparum* (Pf) incidence and maximum humidity in Lao PDR.


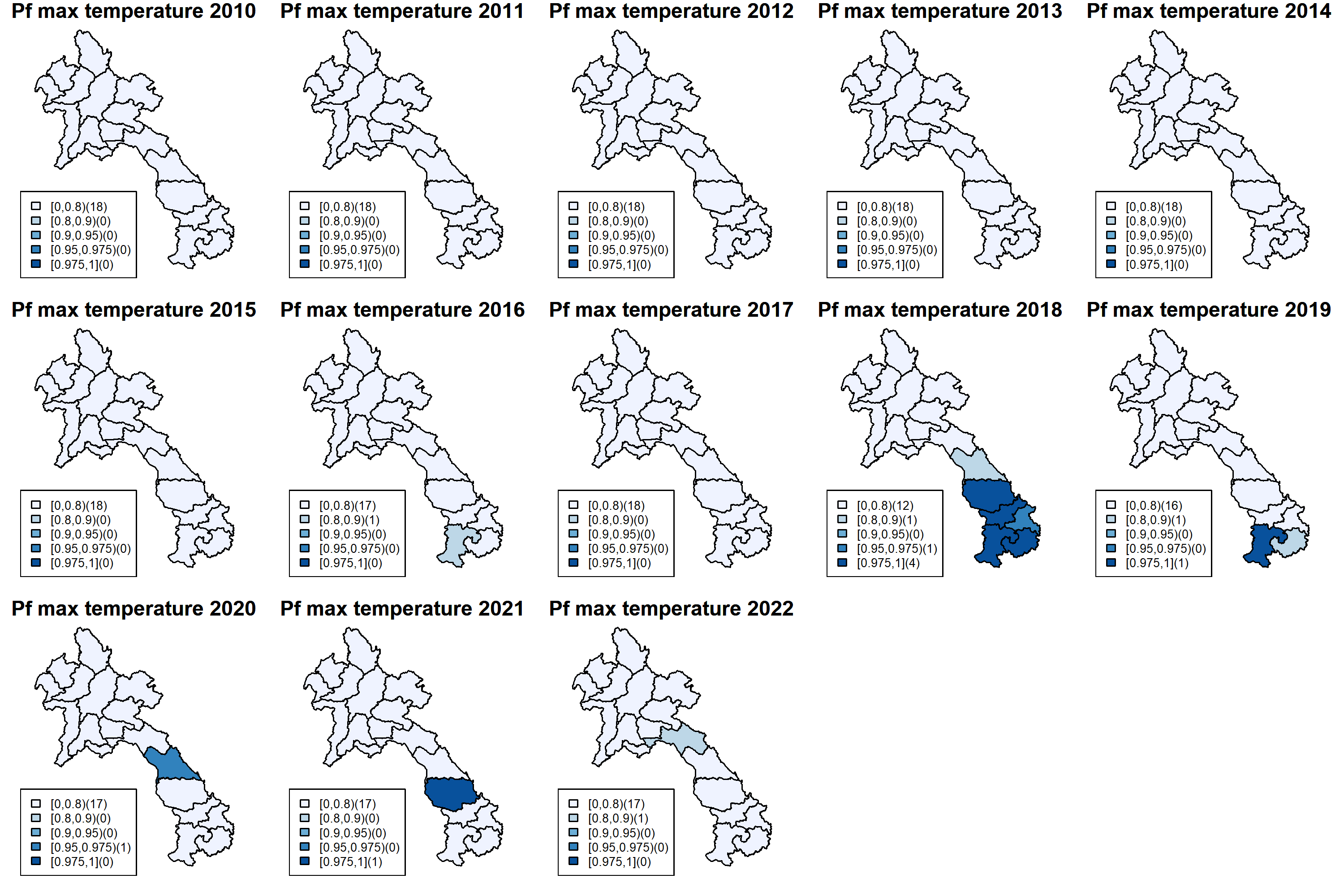


**Figure S3.4** Maps of exceedance probability of association between *P. falciparum* (Pf) incidence and maximum temperature in Lao PDR.


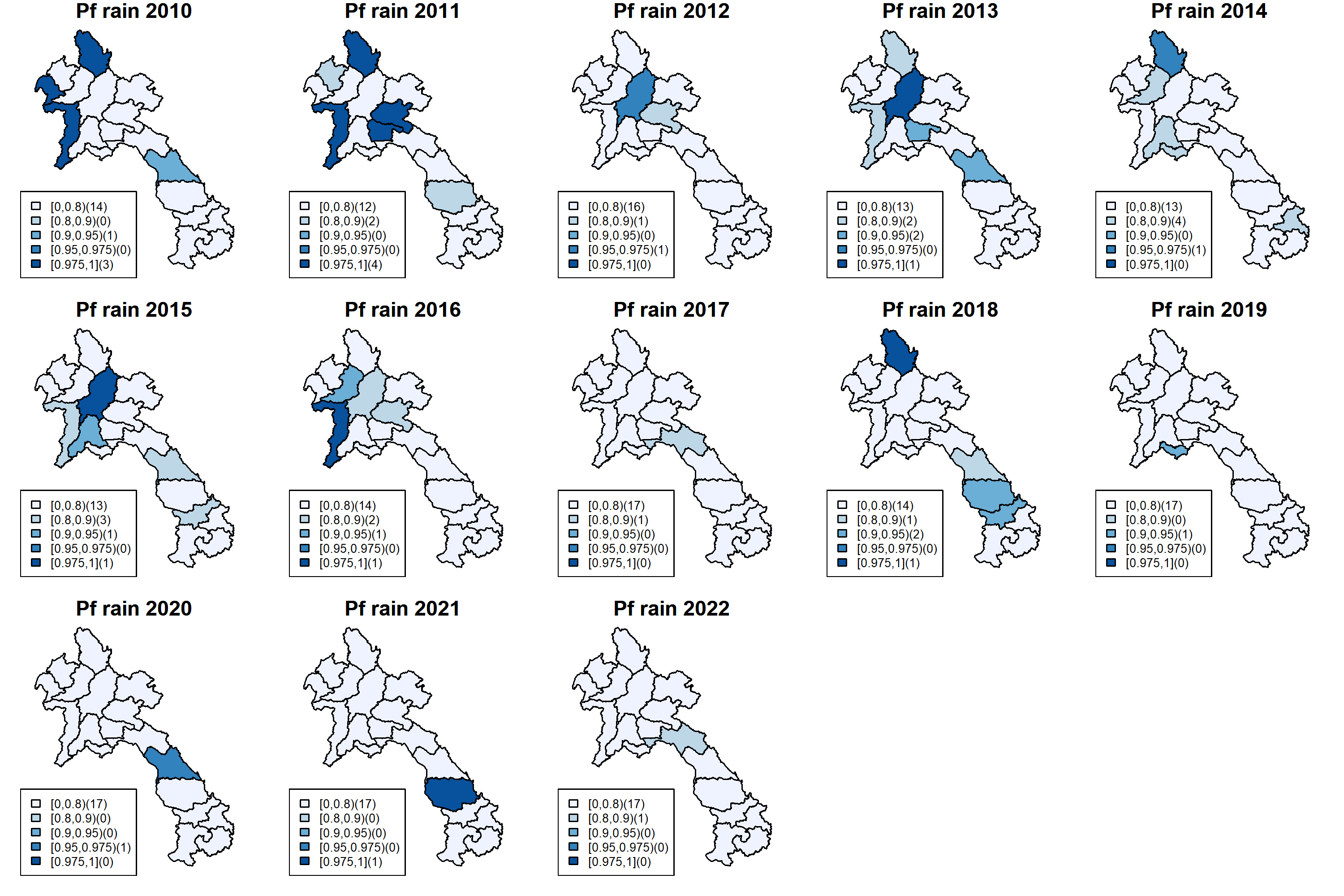


**Figure S3.5** Maps of exceedance probability of association between *P. falciparum* (Pf) incidence and rainfall in Lao PDR.


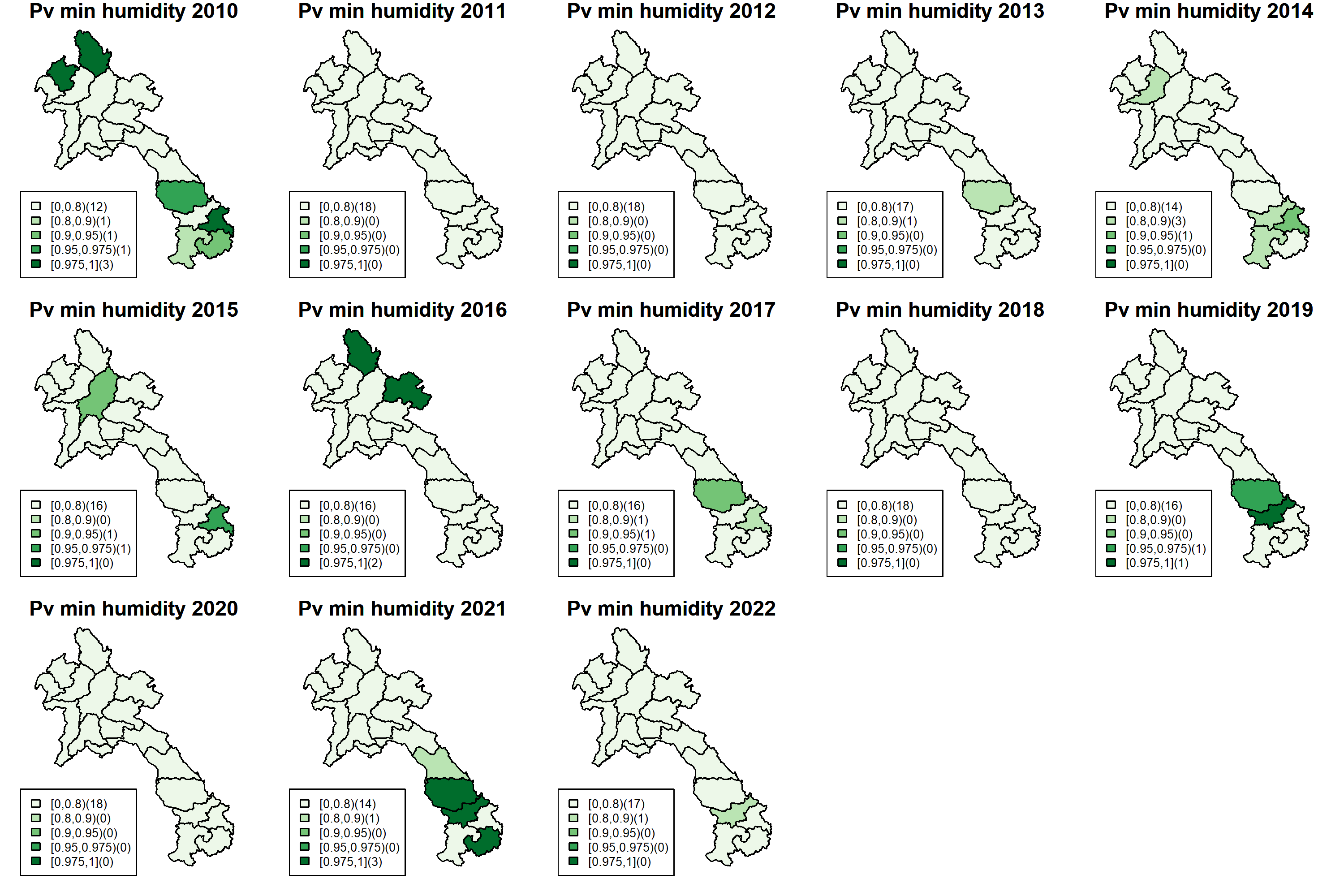


**Figure S3.7** Maps of exceedance probability of association between *P. vivax* (Pv) incidence and minimum humidity in Lao PDR.


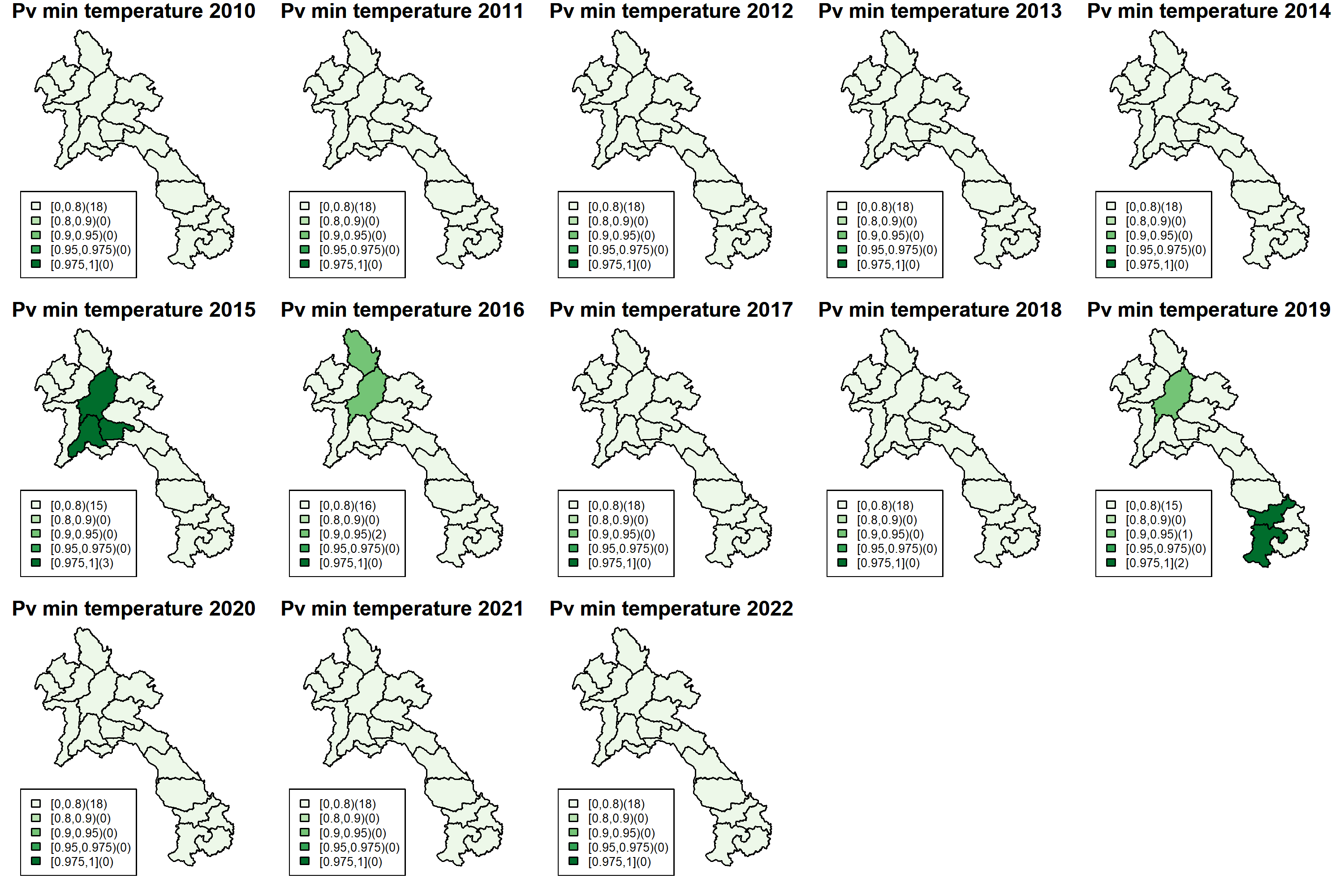


**Figure S3.8** Maps of exceedance probability of association between *P. vivax* (Pv) incidence and minimum temperature in Lao PDR.


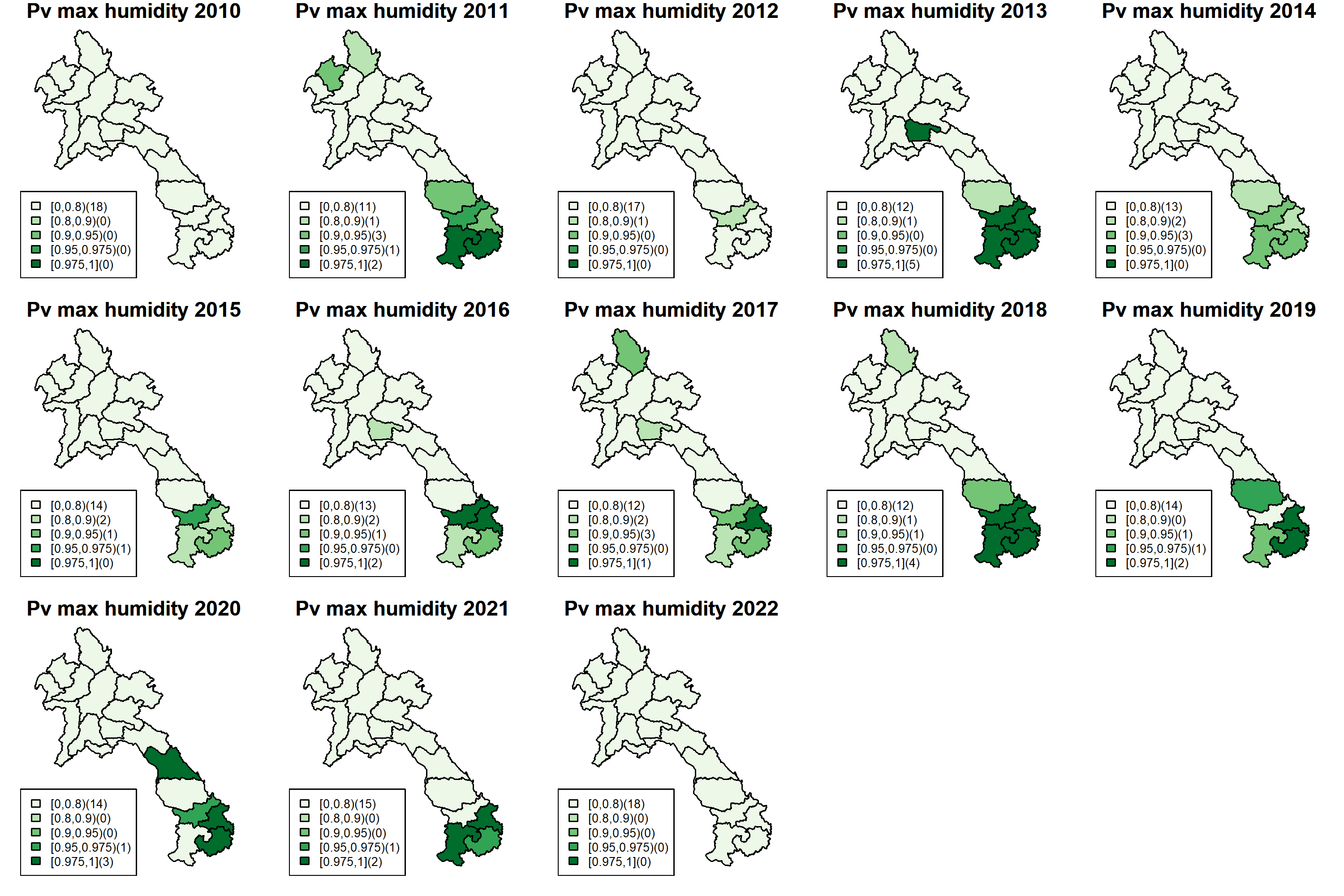


**Figure S3.9** Maps of exceedance probability of association between *P. vivax* (Pv) incidence and maximum humidity in Lao PDR.


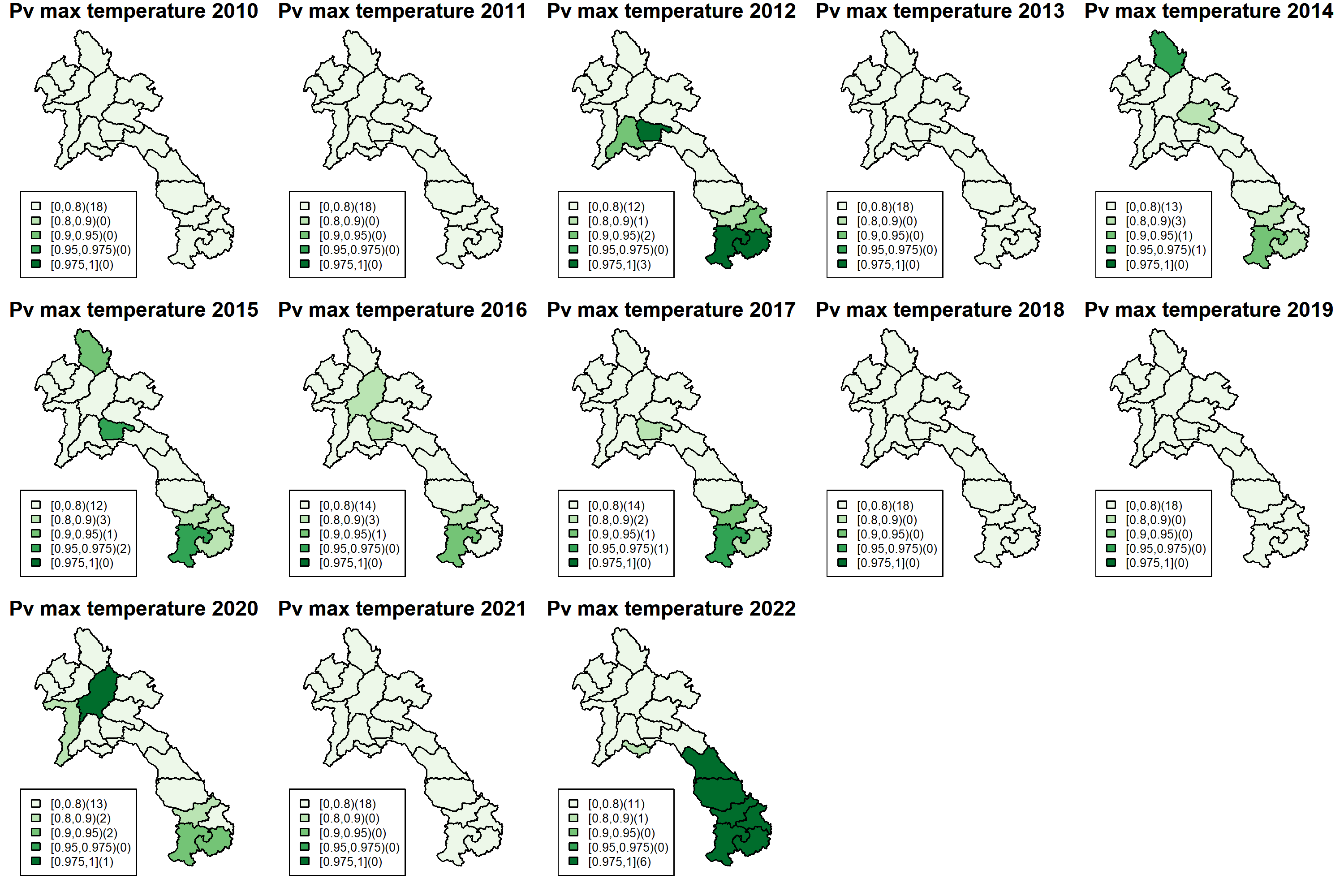


**Figure S3.10** Maps of exceedance probability of association between *P. vivax* (Pv) incidence and maximum temperature in Lao PDR.


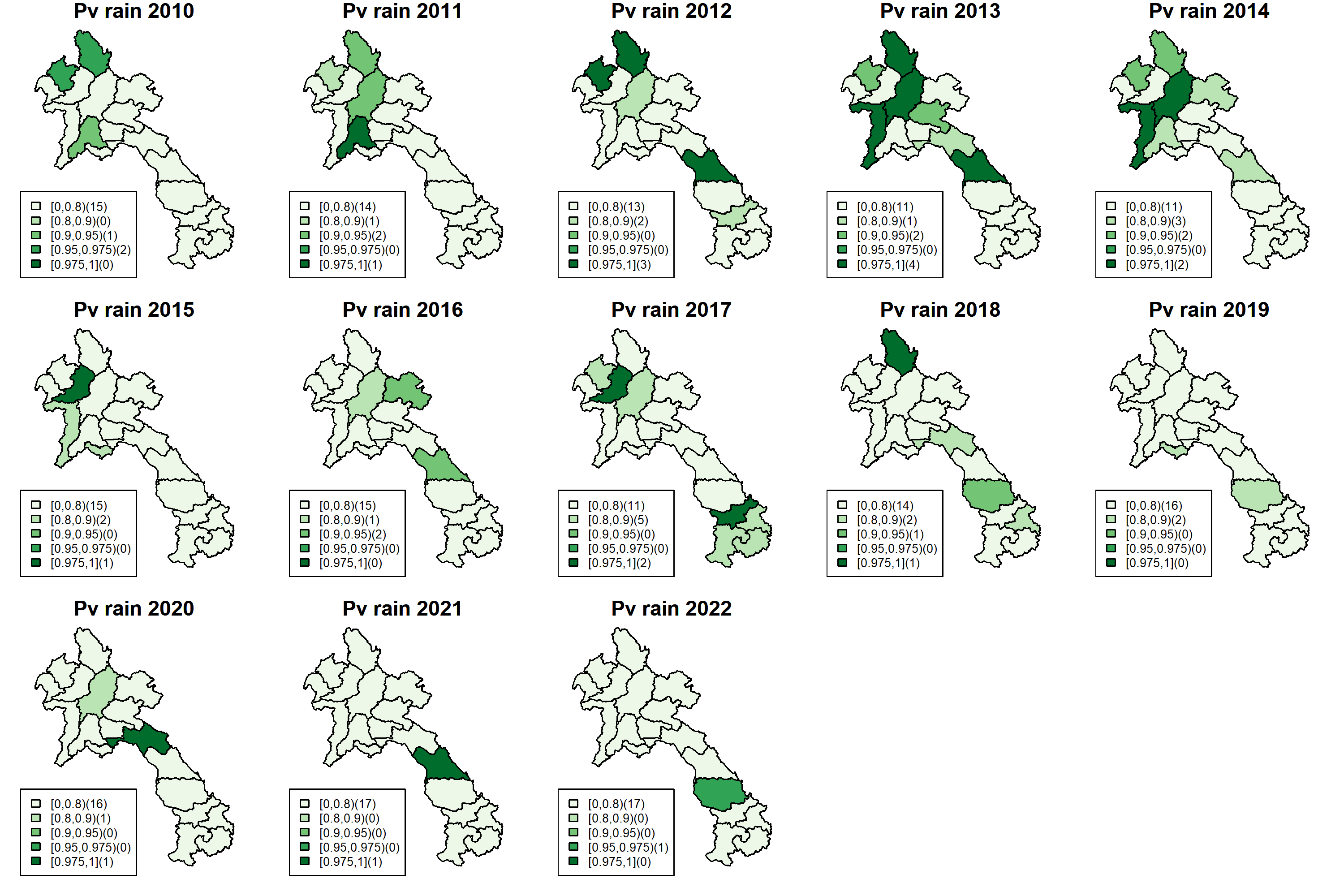


**Figure S3.12** Maps of exceedance probability of association between *P. vivax* (Pv) incidence and rainfall in Lao PDR.

**S4: Tables of incidence rate ratio of *P. falciparum* (Pf) malaria incidence associated with climatic factors during the study period in Lao PDR.**

**S5: Tables of incidence rate ratio of *P. vivax* (Pv) malaria incidence associated with climatic factors during the study period in Lao PDR.**

**S6 Table of climatic factor summary during the study period in Lao PDR.**

| **Climate** | **Min. 1st** | **1st Qu.** | **Median** | **Mean** | **3rd Qu.** | **Max.** |
| --- | --- | --- | --- | --- | --- | --- |
| Rain | 0 | 16.95 | 88.05 | 151.21 | 224.85 | 988.5 |
| Minimum humidity | 0 | 45.83 | 54.77 | 54.69 | 65.42 | 96.11 |
| Minimum temperature | -0.4 | 17.13 | 21.23 | 20.13 | 24.25 | 32.02 |
| Maximum humidity | 0 | 91.26 | 94.83 | 93.39 | 96.81 | 118.6 |
| Maximum temperature | 0 | 29 | 31.5 | 30.58 | 33.36 | 41.2 |

**References**

1. Besag, J., J. York, and A. Mollié, *Bayesian image restoration, with two applications in spatial statistics.* Annals of the institute of statistical mathematics, 1991. **43**(1): p. 1-20.

2. Spiegelhalter, D.J., et al., *Bayesian measures of model complexity and fit.* Journal of the royal statistical society: Series b (statistical methodology), 2002. **64**(4): p. 583-639.

3. Watanabe, S. and M. Opper, *Asymptotic equivalence of Bayes cross validation and widely applicable information criterion in singular learning theory.* Journal of machine learning research, 2010. **11**(12).

4. Vehtari, A., A. Gelman, and J. Gabry, *Practical Bayesian model evaluation using leave-one-out cross-validation and WAIC.* Statistics and computing, 2017. **27**(5): p. 1413-1432.

5. Pettit, L., *The conditional predictive ordinate for the normal distribution.* Journal of the Royal Statistical Society: Series B (Methodological), 1990. **52**(1): p. 175-184.

6. Rotejanaprasert, C., et al., *Bayesian spatio-temporal distributed lag modeling for delayed climatic effects on sparse malaria incidence data.* BMC medical research methodology, 2021. **21**(1): p. 1-15.

7. Rotejanaprasert, C., et al., *Spatiotemporal distributed lag modelling of multiple Plasmodium species in a malaria elimination setting.* Statistical Methods in Medical Research, 2021. **30**(1): p. 22-34.
